# Allergen Content of Therapeutic Preparations for Allergen-Specific Immunotherapy of European Paper Wasp Venom Allergy

**DOI:** 10.3390/toxins14040284

**Published:** 2022-04-15

**Authors:** Johannes Grosch, Antoine Lesur, Stéphanie Kler, François Bernardin, Gunnar Dittmar, Elisabetta Francescato, Simon J. Hewings, Constanze A. Jakwerth, Ulrich M. Zissler, Matthew D. Heath, Markus Ollert, Matthias F. Kramer, Christiane Hilger, Maria Beatrice Bilò, Carsten B. Schmidt-Weber, Simon Blank

**Affiliations:** 1 Center of Allergy and Environment (ZAUM), Technical University of Munich, School of Medicine and Helmholtz Center Munich, German Research Center for Environmental Health, Member of the German Center of Lung Research (DZL), Member of the Immunology and Inflammation Initiative of the Helmholtz Association, 85764 Munich, Germany; johannes.grosch@helmholtz-muenchen.de (J.G.); constanze.jakwerth@tum.de (C.A.J.); ulrich.zissler@tum.de (U.M.Z.); csweber@tum.de (C.B.S.-W.); 2 Quantitative Biology Unit, Luxembourg Institute of Health (LIH), 1445 Strassen, Luxembourg; antoine.lesur@lih.lu (A.L.); francois.bernardin@lih.lu (F.B.); gunnar.dittmar@lih.lu (G.D.); 3 Department of Infection and Immunity, Luxembourg Institute of Health (LIH), 4354 Esch-Sur-Alzette, Luxembourg; stephanie.kler@lih.lu (S.K.); markus.ollert@lih.lu (M.O.); christiane.hilger@lih.lu (C.H.); 4 Entomon SRL, 50012 Florence, Italy; francescato@entomon.it; 5 Allergy Therapeutics PLC, Worthing BN14 8SA, UK; simon.hewings@allergytherapeutics.com (S.J.H.); matthew.heath@allergytherapeutics.com (M.D.H.); kramerm@bencard.com (M.F.K.); 6 Department of Dermatology and Allergy Center, Odense Research Center for Anaphylaxis, University of Southern Denmark, 5000 Odense, Denmark; 7 Bencard Allergie GmbH, 80804 Munich, Germany; 8 Department of Clinical and Molecular Sciences, Polytechnic University of Marche, Ancona and Allergy Unit, Department of Internal Medicine, University Hospital of Ancona, 60121 Ancona, Italy; m.b.bilo@staff.univpm.it

**Keywords:** allergen-specific immunotherapy, American *Polistes* species, European paper wasp, European *Polistes* species, Hymenoptera venom allergy, *Polistes dominula*, therapeutic venom extract, venom immunotherapy

## Abstract

Allergy to *Polistes dominula* (European paper wasp) venom is of particular relevance in Southern Europe, potentially becoming a threat in other regions in the near future, and can be effectively cured by venom immunotherapy (VIT). As allergen content in extracts may vary and have an impact on diagnostic and therapeutic approaches, the aim was to compare five therapeutic preparations for VIT of *P. dominula* venom allergy available in Spain. Products from five different suppliers were analyzed by SDS-PAGE and LC-MS/MS and compared with a reference venom sample. Three products with *P. dominula* venom and one product with a venom mixture of American *Polistes* species showed a comparable band pattern in SDS-PAGE as the reference sample and the bands of the major allergens phospholipase A1 and antigen 5 were assignable. The other product, which consists of a mixture of American *Polistes* species, exhibited the typical band pattern in one, but not in another sample from a second batch. All annotated *P. dominula* allergens were detected at comparable levels in LC-MS/MS analysis of products containing *P. dominula* venom. Due to a lack of genomic information on the American *Polistes* species, the remaining products were not analyzed by this method. The major *Polistes* allergens were present in comparable amounts in the majority, but not in all investigated samples of venom preparations for VIT of *P. dominula* venom allergy.

## 1. Introduction

Venom allergies with their potentially fatal consequences are an important focus in the field of allergy research [1,2,3]. In addition to the general interest in traditional stinging insects of Western and Central Europe, honeybee (*Apis mellifera*) and common wasp (*Vespula vulgaris/germanica*), research interest in species from warmer regions of Europe such as *Polistes dominula*, increased [4,5,6]. *P. dominula* has not only moved from southern to northern Europe in recent decades due to changing climatic conditions, but has also become invasive in several countries around the globe [7,8,9,10,11]. Due to the occupation of new habitats and increased contact with humans [12], *P. dominula* gains in relevance in the context of allergy.

**Figure 1:**
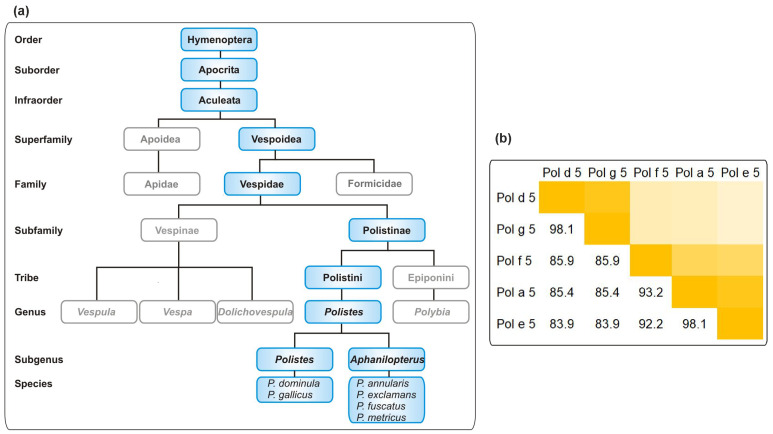
Allergy-relevant *Polistes* species and their relationship. (**a**) Taxonomy of *Polistes* species currently listed in the WHO/IUIS Allergen nomenclature database As the taxonomy of the order Hymenoptera is highly complex, only a selection of allergy-relevant taxa is shown. (**b**) Percentage of identity on protein level between antigen five allergens from allergy-relevant *Polistes* species. The color code ranges from light (low identity) to dark yellow (high identity). Sequence identifiers: Pol d 5: P81656; Pol g 5: P83377; Pol a 5: Q05109; Pol e 5: P35759; Pol f 5: P35780.

The order Hymenoptera is subdivided into the suborders Symphyta and Apocrita. The latter contains the two allergy-relevant superfamilies, Apoidea and Vespoidea. The Vespoidea can be further divided into the allergy-relevant families of the Vespidae (wasps) and the Formicidae (ants). Moreover, the Vespidae contain the subfamilies Vespinae, including the genera *Vespula* and *Vespa*, and Polistinae. The allergy-relevant species from the genus *Polistes* primarily include *P. dominula* and *P. gallicus* (subgenus *Polistes*) on the one hand and, amongst others, *P. annularis*, *P. exclamans*, *P. fuscatus*, and *P. metricus* (subgenus *Aphanilopterus*) on the other hand (Figure 1a) [3,13]. While these members of the subgenus *Aphanilopterus* have their habitat primarily in Northern America [7,14,15], *P. dominula* is found worldwide due to its high invasiveness [7,8,9,10,11].

Robust data on the relevance of *P. dominula* venom allergy are scarce and difficult to collect due to complex diagnostics regarding cross-reactivity to other Vespidae. Studies from Italy and Spain suggest that, at least in the European-Mediterranean area, 34–56% of patients with an allergic reaction after Vespidae stings, had a primary sensitization to *P. dominula* [16,17,18].

However, provided that a patient can be successfully and unambiguously diagnosed, venom allergy can be effectively treated by venom immunotherapy (VIT) [19]. Besides individual characteristics of each patient, the success of VIT is fundamentally dependent on choosing the appropriate venom based on a comprehensive diagnostic workup [3,20].

In addition, the allergen content of therapeutic venom preparations and sensitization profiles of individual patients may have an impact on therapeutic success [21]. Only selected extract-based allergen-specific immunotherapies have been confirmed to be safe and efficacious in clinical trials according to European Directive 2001/83/EC [22]. Although the use of allergen extracts in immunotherapy has been common practice for several decades, no standardized extraction or quality process has been established. In the past, treatment failure in individual patients has been associated with different composition and underrepresented allergens in therapeutic products [21,23,24]. Reports of varying allergen compositions of, e.g., honeybee venom therapy preparations, especially with regard to Api m 10 content [21,25,26], but also of grass pollen and mite extracts [27,28], make a detailed and independent qualitative analysis of therapeutic preparations for allergen-specific immunotherapy desirable. Venom extracts for VIT are drugs and, therefore, need consistent high-quality control and batch-to-batch constant characteristics to ensure safety and efficacy of VIT. In addition, allergen extracts can also be used in diagnostics, for example, for basophil activation tests [29,30]. Here, too, standardized high quality is indispensable. 

Therefore, this study aimed to compare the composition of *P. dominula* VIT products from different manufacturers available on the Spanish market to give insights on the suitability of said medicinal formulations.

Commercially available products for P. dominula venom allergy immunotherapy from five different manufacturers were analyzed: ALK-Abelló (Pharmalgen^®^), Allergy Therapeutics (Venom ATL Polistes^®^; in the following: Venom ATL^®^), Diater Pharmaceutical Laboratory (Diater Veneno de Himenópteros Polistes spp^®^; in the following: Diater veneno^®^), LETI Pharma (Venomvac^®^), and ROXALL Medicina (Hymnox^®^). All products are marketed for the treatment of *P. dominula* venom allergy and are available on the Spanish market. In addition, Entomon Capillary Extracted Venom^®^ *P. dominula* (in the following: Entomon), on which the previously published *P. dominula* venom proteome is based [5], was analyzed as the reference venom. The products Diater veneno^®^ and Venomvac^®^ do not contain *P. dominula* venom, but a mixture of the venoms of American *Polistes* species. According to LETI Pharma, Venomvac^®^ contains venom of the species *P. fuscatus*, *P. metricus*, *P. exclamans*, and *P. annularis*, while Diater Pharmaceutical Laboratory states that Diater veneno^®^ comprises the venoms of various species of *Polistes* such as *fuscatus*, *metricus*, *annularis*, and *exclamans*.

## 2. Results

Commcercially available products (Table 1) were compared for their composition by SDS-PAGE and LC-MS/MS.

### 2.1. LC-MS/MS of P. dominula Venom-Containing Venom Preparations

European and American *Polistes* species belong to different subgenera (Figure 1a) [15]. The degree of relatedness of the individual species influences the sequence similarity of the venom allergens. Figure 1b shows exemplarily a sequence identity matrix for annotated Antigen 5 (Ag5) allergens from *P. dominula*, *P. gallicus*, *P. fuscatus*, *P. exclamans*, and *P. annularis*. While the closely related *P. dominula* and *P. gallicus* show Ag5 identity of around 98%, more distantly related American species such as *P. exclamans* show an identity of 84% to *P. dominula* Ag5 [13].

**Figure 2:**
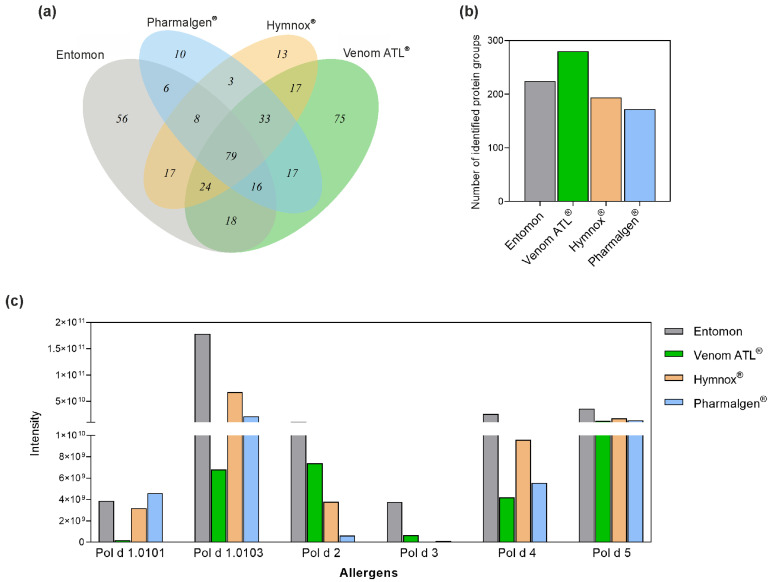
Analysis of *P. dominula* venom-containing therapeutic venom preparations by LC-MS/MS. (**a**) Number of unique protein groups of the preparations and the overlap regions containing proteins identified in different preparations. (**b**) Total number of protein groups detected. (**c**) Quantitative comparison of the amounts of annotated *P. dominula* venom allergens. Protein groups contain proteins sharing the same identified peptides. Protein intensities were calculated from the sum of the respective peptide intensities corresponding to the areas of the extracted ion chromatograms of the peptides. ^®^, registered trademark.

The LC-MS/MS analyses were only performed for Pharmalgen^®^, Venom ATL^®^, Hymnox^®^, and the reference venom from Entomon as no genomes matching the peptides of the aforementioned American species have been published. Figure 2a shows a Venn diagram of the protein groups identified by mass spectrometry in these products. All products share a cut set of 79 protein groups, which also includes the annotated allergens from *P. dominula* [31,32]. The capillary-extracted venom shows 56 product-specific protein groups, Pharmalgen^®^ 10, Hymnox^®^ 13, and Venom ATL^®^ 75.

The protein diversity is 172 (Pharmalgen^®^), 194 (Hymnox^®^), 224 (Entomon), and 280 (Venom ATL^®^) protein groups (Figure 2b). Therefore, the products contain up to 100 additional proteins, which is probably due to differences in the extraction and manufacturing process. However, the majority of these proteins represents household contaminants or venom trace molecules from cells of the venom gland and its surrounding tissue with potentially negligible allergological relevance (Table S1).

In addition, the protein content of the annotated *P. dominula* allergens in the products from Entomon, Allergy Therapeutics, ALK-Abelló, and ROXALL Medicina was quantitatively compared using LC-MS/MS (Figure 2c). Annotated *P. dominula* allergens are phospholipase A1 (Pol d 1), hyaluronidase (Pol d 2), dipeptidyl peptidase IV (Pol d 3), serine protease (Pol d 4), and antigen 5 (Pol d 5) [31,32,33].

Peptides of two different isoforms of Pol d 1, Pol d 1.0101 and Pol d 1.0103, were found. These two isoforms have 100% query coverage and 99% sequence identity (314/316 amino acids), so they differ only minimally. Hymnox^®^ and Pharmalgen^®^ contained Pol d 1 in comparable amounts, regardless of isoform. Venom ATL^®^ showed a slightly lower Pol d 1 content in these measurements, while Entomon’s reference venom contained more than the three therapy products.

The opposite picture emerged for Pol d 2 and Pol d 3. Although the capillary-extracted venom contained the highest amounts, the levels of these two allergens were higher in Venom ATL^®^ compared to the other therapy products. Hymnox^®^ contained more Pol d 2 than Pharmalgen^®^, while both Pharmalgen^®^ and Hymnox^®^ contain the least amount of Pol d 3.

All therapy products contained Pol d 4 and the major allergen Pol d 5 in comparable amounts. Only the reference venom contained more of these proteins (Figure 2c).

As an intrinsic control, the products were analyzed for the presence of the 12 ‘true venom components’ (secreted proteins and proteins with known function in venom that are not annotated as allergens), which have been published based on proteomic analysis of capillary-extracted venom from Entomon [5]. Eight out of these 12 protein groups were again detected in the venom from Entomon, four in Venom ATL^®^, and five each in Hymnox^®^ and Pharmalgen^®^ (Figure S1). Only dominulin-B was detectable exclusively in capillary-extracted venom from Entomon, probably as this 17 amino acid peptide is lost during the manufacturing process of the other products. 

### 2.2. SDS-PAGE Analysis of Polistes Venom Preparations

**Figure 3:**
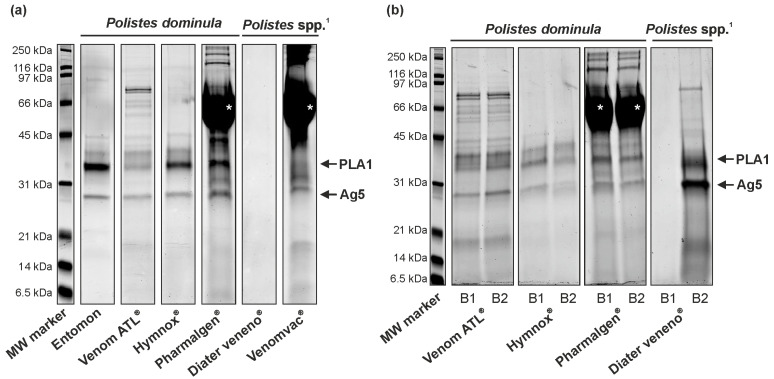
SDS-PAGE analysis of *Polistes* venom preparations. (**a**) Comparison of five therapeutic preparations and a reference venom sample. (**b**) Comparison of two batches of four of the therapeutic preparations. White asterisks indicate the band of HSA contained in some of the preparations. ^1^ *Polistes* spp.: venom mixture from American *Polistes* species. Ag5, antigen 5; B, batch; PLA1, phospholipase A1. ^®^, registered trademark.

Figure 3a shows strips from an SDS-PAGE of a product batch of the Entomon reference venom, Venom ATL^®^, Hymnox^®^, Pharmalgen^®^, Diater veneno^®^, and Venomvac^®^. A total of 15 µg of venom (based on the manufacturer’s specifications for the products) were applied under reducing conditions per sample. The therapy products were diluted in 1/10 of the actual recommended amount (for VIT injections) to bring the concentration of the samples to a detectable level. Clear and distinct bands of phospholipase A1 (PLA1) and antigen 5 (Ag5) can be discerned for the capillary-extracted venom from Entomon, Venom ATL^®^, and Hymnox^®^. Pharmalgen^®^ and Venomvac^®^ contain human serum albumin (HSA) in the lyophilized venom, resulting in a prominent band between 50 to 70 kDa obscuring some less prominent bands. However, the expected bands of PLA1 and Ag5 are also evident here. Diater veneno^®^ shows no bands. 

For quality control and to ensure that no staining method-, laboratory-, or operator-specific factor influenced the results, a second vial of these batches from the same package was analyzed by SDS-PAGE using a different staining method in a different laboratory by a different operator. This second analysis confirmed the previous results (Figure S2). All products were stored according to the manufacturer’s instruction and the cold chain was not interrupted. To further confirm the findings and to describe possible batch-to-batch variability, the manufacturers were asked to provide a different batch of their respective products. The second batches are shown in Figure 3b in comparison to the first batch for all manufacturers except LETI Pharma. Venom ATL^®^, Hymnox^®^, and Pharmalgen^®^ showed similar results in the banding pattern of the two batches. Therefore, batch-to-batch variability, with regard to the main allergens as detected in SDS-PAGE, appears to be low. However, one product presented a different result and must be considered separately: the two batches of Diater veneno^®^ displayed different patterns. While not a single band was detectable in the SDS-PAGE of the first batch, the bands for PLA1 and Ag5 could be detected in the second batch. This Diater veneno^®^ batch has a particularly prominent Ag5 band, which indicates a higher content of this specific allergen compared to the other products.

### 2.3. LC-MS of Polistes Venom Preparations

**Figure 4:**
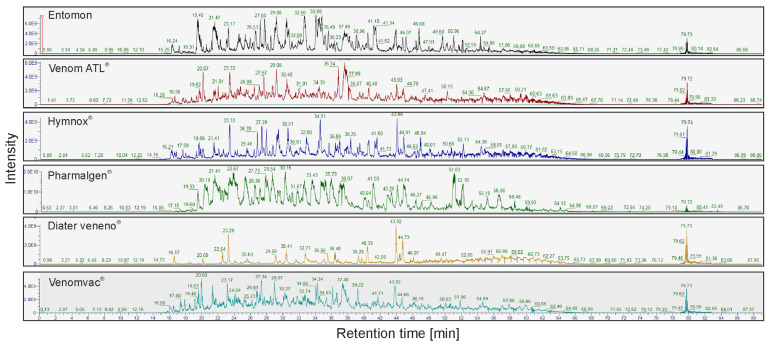
LC-MS analysis of *Polistes* venom preparations. Total ion chromatograms of the LC-MS analysis of five different therapeutic preparations and a reference venom sample.

LC-MS was used to confirm the results from the SDS-PAGEs of the first batches, in particular of Diater veneno^®^ (Figure 4). While the intensity and richness of the peaks were comparable for Entomon, Pharmalgen^®^, Venom ATL^®^, Venomvac^®^, and Hymnox^®^, the analyzed product of Diater showed a different picture, i.e., fewer and less pronounced peaks. This suggests that the first batch of Diater veneno^®^ tested contained hardly any analyzable protein.

## 3. Discussion

VIT is the only available curative approach for the treatment of Hymenoptera venom allergy and is effective in the majority of allergic patients [19]. Nevertheless, differences in the allergen content of therapeutic preparations for VIT of honeybee venom allergy have recently been demonstrated [21,25,26,34]. However, to date, it remains unknown whether such differences in allergen composition contribute to the observed lower efficacy of VIT in patients with particular sensitization profiles [21]. There are no published data on the allergen composition and content of therapeutic *Polistes* venom preparations. To characterize product composition, SDS-PAGEs and LC-MS/MS analyses of five products marketed for *P. dominula* venom allergy were performed.

Although the number of protein groups identified in the different venom samples is in a comparable range, differences in diversity can be attributed in part to the extraction and manufacturing process. According to the manufacturers, Venom ATL^®^ is extracted from homogenized venom glands, while Hymnox^®^ is derived from venom of squeezed glands. For Pharmalgen^®^, hand-dissected venom sacs are homogenized using a tissue grinder. According to the current state of research and available clinical data, it is not to be expected that the relatively small differences in protein diversity will have a significant impact on the success of VIT.

Many of the closely related Vespoidea species share the same four allergens: phospholipase A1, hyaluronidase, dipeptidyl peptidase IV, and antigen 5 [33]. For *P. dominula*, these proteins were annotated as Pol d 1 (34 kDa), Pol d 2 (50 kDa), Pol d 3 (100 kDa), and Pol d 5 (23 kDa). Additionally, the serine protease Pol d 4 (33 kDa) is annotated in the WHO/IUIS database [31,32]. The homologous allergens of closely related Hymenoptera species usually show very similar behavior in SDS-PAGE [35]. It can, therefore, be expected that the allergens from the products with mixed venoms from American *Polistes* species will exhibit a similar band pattern compared to the annotated allergens from *P. dominula*. Since Pol d 1 and Pol d 4 have similar molecular weights, differentiation in normal SDS-PAGEs is not expected. Assignable in the SDS-PAGEs are the bands for the major allergens phospholipase A1 and antigen 5 in the reference venom from Entomon, as well as in Venom ATL^®^, Pharmalgen^®^, and Hymnox^®^. Venomvac^®^ shows a clear band for antigen 5, but phospholipase A1 is not clearly visible, probably because of the HSA content. Diater veneno^®^ shows both bands; however, only in the sample from the second batch analyzed. Hyaluronidase and dipeptidyl peptidase IV are either not visible in the SDS-PAGEs or are difficult to assign due to the low concentration in the venom. However, the band of approximately 100 kDa, clearly visible in the second batch of Diater veneno^®^ and slightly visible in the venom from Entomon, could be assigned to dipeptidyl peptidase IV. In the ALK-Abelló and LETI Pharma products, the high HSA content, resulting in a very prominent band in the region of interest, interferes with appropriate detection of allergens in this molecular weight range by SDS-PAGE. Nevertheless, the analyses clearly demonstrate that the most relevant major allergens PLA1 and Ag5 are present in all products, although variability was demonstrated in samples of Diater veneno^®^ from two different batches. Fluctuations in the content of particular allergens in different batches of a product have been also demonstrated for therapeutic honeybee venom preparations before [25,34]. 

The work presented here should be considered in the overall context of the research already conducted on the heterogeneity of allergen extracts for immunotherapy. It has already been shown that the allergen content of honeybee [21,25,26], birch pollen, mite, [27,36], dog [37], and timothy grass [28] extracts varies. Unlike honeybee or dog allergen extracts, for example, no completely absent major allergens could be identified for the *P. dominula* preparations analyzed here. Thus, the tested products are in, principle, all suitable for immunotherapy of the majority of *P. dominula* venom allergic patients. However, the question of consistent batch-to-batch quality may be a problem.

Although it is an encouraging result that the major *Polistes* venom allergens are detectable in the majority of the analyzed VIT preparations available on the Spanish market for the treatment of European *Polistes* venom allergy, there is controversy as to whether preparations containing pure *P. dominula* venom and venom preparations containing mixtures of American *Polistes* species are equally suitable for treating European patients, primarily sensitized to European species. An early Spanish study showed that the majority of sera from patients sensitized to *P. dominula* venom were also positive for the closely related *P. gallicus* venom [38]. In addition, comparison of these two European species with different American species showed that most patients were positive for all species, although there were significant differences in the radioallergosorbent test (RAST) value. The authors confirmed these results through RAST inhibition studies and concluded that European and American *Polistes* species are closely related, but species-specific allergenic differences exist. Another study from Italy also performed RAST inhibition experiments and demonstrated that cross-reactivity between European and American *Polistes* species is only partial and that the European species have exclusive allergens [39]. These results were confirmed by skin testing and direct RAST. It is worth noting that the four patients who were negative in the intradermal test for American *Polistes* species were positive for *P. dominula* and *P. gallicus*. The authors conclude that European *Polistes* venom is more suitable for VIT than the American mix in Italian patients and that European *Polistes* venoms are necessary for diagnosis and therefore also for therapy of European patients. In addition, a case report showed that treatment with American *Polistes* spp. venom was ineffective in an Italian patient allergic to European *Polistes* species, while the patient was successfully treated with *P. dominula* venom [40]. Furthermore, a Spanish patient on VIT with the American *Polistes* species venom mixture developed anaphylaxis following a *P. dominula* sting challenge, while no reaction occurred with the same sting challenge when treatment was switched to the *P. dominula* extract after a more sensitive diagnosis using rPol d 1-specific IgE [41]. Interestingly, a recent retrospective study from Italy compared the efficacy of VIT with *P. dominula* venom (102 patients) and a mixture of American *Polistes* species (89 patients) and found that clinical protection against in-field stings was statistically comparable between the two groups [42]. However, only patients with a double-sensitization to European and American *Polistes* species were analyzed in this study and mono-sensitization to *P. dominula* was an exclusion criterion. The authors conclude that there is no scientific report supporting the exclusive use of *P. dominula* venom for VIT in patients with *Polistes* venom allergy unless the patient is mono-sensitized to *P. dominula* venom. Finally, prospective studies are needed to answer the question of which venom preparations are most suitable for the treatment of European *Polistes* venom allergy. 

In the present study, the allergen content of the products containing *P. dominula* venom was quantitatively compared using LC-MS/MS. Due to a lack of genomic information for American *Polistes* species, the products containing venom mixtures of those species could not be analyzed using this method. It is striking that the capillary-extracted venom from Entomon delivers by far the highest values for all allergens. In addition to the gentle extraction process, this can probably be attributed to the minimal post-procession. The three manufacturers of immunotherapy products are all coming up with individual profiles. While the Pol d 1 content in Pharmalgen^®^ and Hymnox^®^ is higher than in Venom ATL^®^, the opposite picture applies to Pol d 2 and Pol d 3. All therapy products provide overall comparable values for Pol d 4 and Pol d 5, with Hymnox^®^ as the leader by a small margin.

## 4. Conclusions

In summary, it can be stated that the three pure *P. dominula* venom-containing products compared are probably all equally well suited for VIT since they contain the relevant allergens in comparable quantities. None of the products analyzed by mass spectrometry show an obvious weakness in terms of allergen content. According to the manufacturer, Pharmalgen^®^ will be discontinued at the end of 2023. Another product, consisting of a mixture of venoms from American *Polistes* species, shows a similar band pattern in SDS-PAGE indicating the presence of the homologous allergens to Pol d 1 (phospholipase A1) and Pol d 5 (antigen 5) from these species. For the second product containing such a venom mixture, the relevant allergens could be detected by SDS-PAGE in one, but not in a second sample from a different batch. However, translation of this research into clinical practice is somewhat limited by its focus on detectable protein content only. In particular, this study does not look into in vivo immunogenicity of the detected proteins, which is certainly needed and should be addressed in future research. With regard to the safety and efficacy of VIT with *P. dominula* venom, randomized, double-blind, placebo-controlled clinical trials are required to ensure the best treatment chances for patients with venom allergy. Nevertheless, this study demonstrates that the relevant *Polistes* venom allergens were present to a comparable extent in the majority, but not in all examined samples of preparations for VIT of *P. dominula* venom allergy.

## 5. Materials and Methods

### 5.1. Products

A total of six commercially available products were included. Entomon Capillary Extracted Venom^®^ *P. dominula* venom (Entomon) (Entomon, Florence, Italy) served as reference venom, two batches of Venom ATL Polistes^®^ (Venom ATL^®^) (Allergy Therapeutics, Worthing, UK), two batches of Hymnox^®^ (ROXALL Medicina, Zamudio, Spain), two batches of Pharmalgen^®^ (ALK-Abelló, Madrid, Spain), two batches of Diater Veneno de Himenópteros Polistes spp^®^ (Diater veneno^®^) (Diater Pharmaceutical Laboratory, Madrid, Spain), and one batch of Venomvac^®^ (LETI Pharma, Madrid, Spain).

### 5.2. SDS-PAGE 

The lyophilized products were freshly reconstituted with ddH_2_O. A total of 15 µg (based on the manufacturer’s specifications) of venom were applied under reducing conditions to Any kD™ Mini-PROTEAN^®^ TGX™ Precast Protein Gels (Bio-Rad, Munich, Germany) and proteins separated according to their molecular weight. Bands were visualized using SyproRuby (Thermo Fisher Scientific, Waltham, MA, USA) staining and Typhoon scan (350–400 PMT/25 µm) (Cytiva, Marlborough, MA, USA). Samples were loaded and ran on a single SDS-PAGE.

### 5.3. Sample Preparation

Venom protein concentration was measured by bicinchoninic acid assay (Thermo Fisher Scientific) and an aliquot of 10 µg of protein per sample processed with SP3 beads (Thermo Fisher Scientific) following the protocol of Hughes et al. [43]. Briefly, venoms were centrifuged, and the cysteine disulfide bonds were reduced and alkylated with dithiothreitol (40 mM final) and iodoacetamide (120 mM final), respectively. SP3 beads were added to the samples to bind the proteins and the beads were washed three times with 70% ethanol. The beads were then incubated with 0.4 µg of trypsin/lysC mixture (Promega, Madison, WI, USA) in 50 mM ammonium bicarbonate overnight at 37 °C. The peptides formed by the proteolysis were captured onto the beads by adding pure acetonitrile, and the beads were washed three times with acetonitrile before eluting the peptides with 2% DMSO in water. The samples were acidified to pH 4 with formic acid. Samples were vacuum dried and suspended in 15 µL of loading buffer (1% acetonitrile, 0.05% trifluoroacetic acid in water).

### 5.4. Liquid Chromatography-Mass Spectrometry

For the LC-MS/MS analysis, a U3000 RSLC liquid chromatography system from Dionex in column switching mode was used together with a Q-Exactive HF mass spectrometer (Thermo Fisher Scientific). A constant volume of 3 µL per sample was loaded onto a trap column (75 µm × 20 mm, C18 Pepmap 100, 3 µm; Thermo Fisher Scientific) with a loading phase of 1% acetonitrile, 0.05% trifluoroacetic acid in water at a flow rate of 5 µL/min before eluting the samples onto an analytical column (75 µm × 150 mm, C18 Pepmap 100, 2 µm; Thermo Fisher Scientific) with a linear gradient of 2% to 35% solution A in B (solution A: 0.1% formic acid in water; solution B: 0.1% formic acid in acetonitrile). The MS acquisition method was DDA (data-dependent acquisition). A survey scan was performed at 60.000 resolution at 200 *m*/*z* before selection and fragmentation of the 12 most intense precursor ions. The already fragmented precursor ions were excluded for 30 s and the resolution of the MS/MS scan was set to 15.000 at 200 *m*/*z*.

### 5.5. Data Processing

The collected data were processed using MaxQuant 1.6.7.0 [44] and the NCBI *P. dominula* protein database with 21,077 entries. Proteins were identified based on at least two peptides (razor and unique). Protein intensities were calculated from the sum of the respective peptide intensities corresponding to the areas of the extracted ion chromatograms of the peptides. The MS signal is extracted with a very narrow mass window centered on the peptide’s theoretical masses. Due to the high resolution and accuracy of the used mass spectrometer, it is possible to use narrow extraction windows and, thus, remove interferences and peptides of close masses.

## Figures and Tables

**Table 1:** The companies that were included in this study, the name of the corresponding product including the abbreviations used in the running text, and the species included in the preparation. ^1^ *Polistes* spp.: venom mixture from American Polistes species. ^®^, registered trademark.

Company	Product Name	Abbreviation	Species
Entomon	Entomon Capillary Extracted Venom^®^ *P. dominula*	Entomon	*P. dominula*
Allergy Therapeutics	Venom ATL Polistes^®^	Venom ATL^®^	*P. dominula*
ROXALL Medicina	Hymnox^®^	Hymnox^®^	*P. dominula*
ALK-Abelló	Pharmalgen^®^	Pharmalgen^®^	*P. dominula*
Diater Pharmaceutical Laboratory	Diater Veneno de Himenópteros Polistes spp.^®^	Diater veneno^®^	*Polistes* spp.^1^
LETI Pharma	Venomvac^®^	Venomvac^®^	*Polistes* spp.^1^

## Data Availability

All data generated or analyzed during this study are included in this article and its Supplementary Material files.

## Supplementary Materials

The following supporting information can be downloaded at: https://www.mdpi.com/article/10.3390/toxins14040284/s1, Figure S1: Analysis of *P. dominula* venom-containing therapeutic venom preparations by LC-MS/MS; Figure S2: SDS-PAGE analysis of *Polistes* venom-containing therapeutic preparations; Table S1: All identified protein groups including unique identifier for each protein, FASTA headers and intensities.
