# Supplementary material for: Allergen Content of Therapeutic Preparations for Allergen-Specific Immunotherapy of European Paper Wasp Venom Allergy
Source: Toxins (Basel). 2022 Apr 15;14(4):284. doi: 10.3390/toxins14040284 (PMC9031911; doi:10.3390/toxins14040284)
Supplement: Supplementary file 1 [file toxins-14-00284-s001.zip › toxins-1644051-supplementary.pdf]

Supplementary Materials: Allergen Content of Therapeutic Preparations for Allergen-Specific Immunotherapy of European Paper Wasp Venom Allergy

Johannes Grosch, Antoine Lesur, Stéphanie Kler, François Bernardin, Gunnar Dittmar, Elisabetta Francescato, Simon J. Hewings, Constanze A. Jakwerth, Ulrich M. Zissler, Matthew D. Heath, Markus Ollert, Matthias F. Kramer, Christiane Hilger, Maria Beatrice Bilò, Carsten B. Schmidt-Weber and Simon Blank

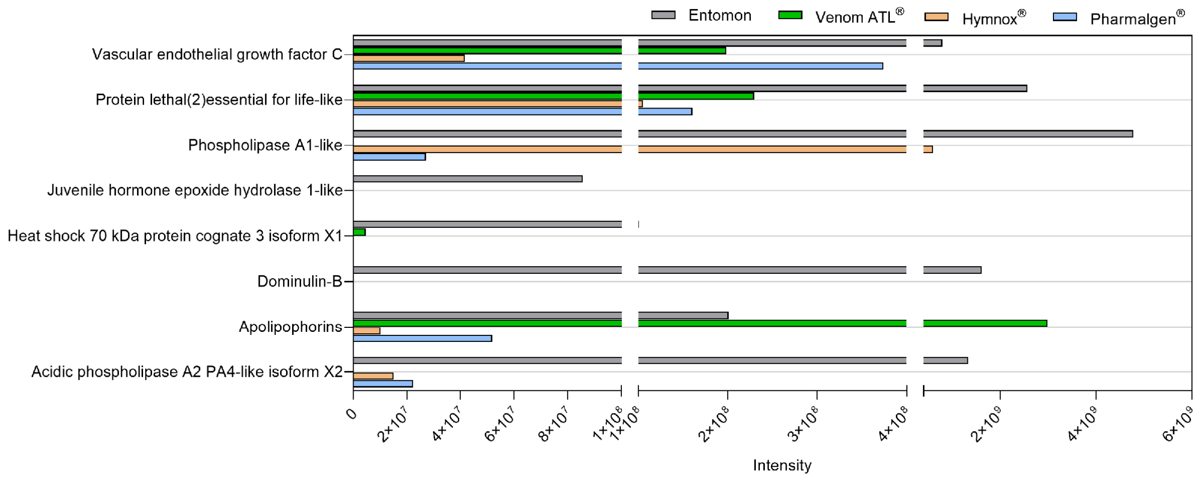

Figure S1. Analysis of *P. dominula* venom-containing therapeutic venom preparations by LC-MS/MS. Quantitative comparison of the amounts of proteins, which are not annotated as allergens, but identified as 'true venom components'.

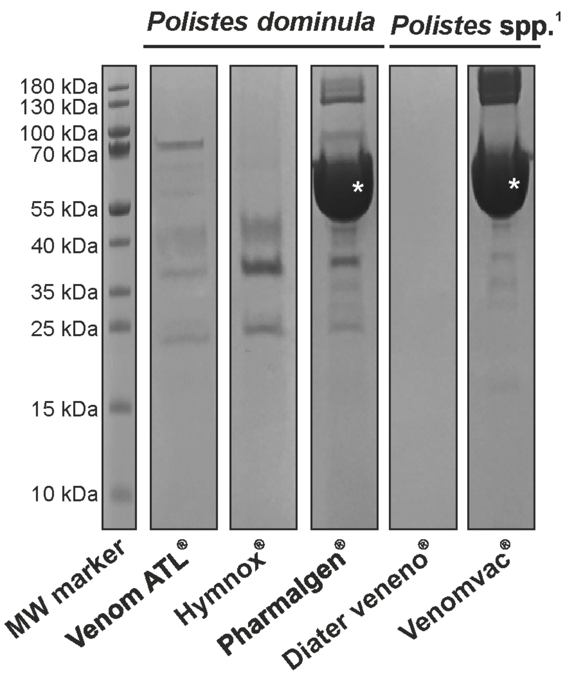

Figure S2. SDS-PAGE analysis of *Polistes* venom-containing therapeutic preparations. Analysis of a second vial from the same package of the batches of five different therapeutic *Polistes* venom preparations analysed in Figure 3a. The analysis was performed in a different laboratory by another operator. The lyophilized products were freshly reconstituted with ddH$_2$0 and a total of 15 µg (according to the specifications of the manufacturers) venom were separated based on their molecular weight using freshly casted Tris-Tricine gels and visualized by Coomassie blue staining. White asterisks indicate the band of human serum albumin contained in some of the venom preparations. [1]*Polistes* spp. venom contains a mix of venoms from different American *Polistes* species such as *P. fuscatus*, *P. metricus*, *P. exclamans*, and *P. annularis*. MW, molecular weight; SDS-PAGE, sodium dodecyl sulfate polyacrylamide gel electrophoresis.

Table S1. All identified protein groups including unique identifier for each protein, FASTA headers and intensities. Protein Ids: unique protein identifier (accession number). Protein group: proteins sharing the same identified peptides. Number of proteins: number of proteins in a protein group. Peptides: number of peptides assigned to one protein group. Razor peptide: a peptide assigned to the protein group with the largest number of total peptides identified. Razor + unique peptides: peptides assigned to the protein group with the largest number of total peptides, which only match to this specific protein group. Unique peptides: unique peptide sequences after removing redundancy from peptide hits.

								Intensity			
**Protein IDs**	**Majority protein IDs**	**Fasta headers**	**Number of proteins**	**Peptides**	**Razor + unique peptides**	**Unique peptides**	**Mol. weight [kDa]**	**Entonom**	**Venom ATL**	**Pharmalgen**	**Hymnox**
XP_015187185.1;sp|Q6Q252.1|PA11_POLDO;AAS67041.1	XP_015187185.1;sp|Q6Q252.1|PA11_POLDO;AAS67041.1	XP_015187185.1 PREDICTED: phospholipase A1 1 [Polistes dominula];sp|Q6Q252.1|PA11_POLDO RecName: Full=Phospholipase A1 1; AltName: Allergen=Pol d 1; Flags: Precursor;AAS67041.1 venom phospholipase A1 1 precursor [Polistes dominula]	3	31	2	0	37,559	3866200000	179370000	4568000000	3174700000
sp|P0C1M7.1|MASTB_POLDO	sp|P0C1M7.1|MASTB_POLDO	sp|P0C1M7.1|MASTB_POLDO RecName: Full=Dominulin-B	1	2	2	2	1,9113	1616400000	0	0	0
XP_015171391.1	XP_015171391.1	XP_015171391.1 PREDICTED: uncharacterized protein YJR142W [Polistes dominula]	1	2	2	2	37,671	0	0	0	0
XP_015171393.1	XP_015171393.1	XP_015171393.1 PREDICTED: aspartate aminotransferase, mitochondrial [Polistes dominula]	1	2	2	2	47,906	16371000	0	0	0
XP_015171471.1	XP_015171471.1	XP_015171471.1 PREDICTED: probable salivary secreted peptide [Polistes dominula]	1	2	2	2	14,523	0	0	0	7722500
XP_015171577.1;XP_015171576.1;XP_015171575.1	XP_015171577.1;XP_015171576.1;XP_015171575.1	XP_015171577.1 PREDICTED: pyruvate carboxylase, mitochondrial isoform X3 [Polistes dominula];XP_015171576.1 PREDICTED: pyruvate carboxylase, mitochondrial isoform X2 [Polistes dominula];XP_015171575.1 PREDICTED: pyruvate carboxylase, mitochondrial isoform	3	2	2	2	112,04	0	0	106590000	0
XP_015171752.1;XP_015171751.1	XP_015171752.1;XP_015171751.1	XP_015171752.1 PREDICTED: phosphoserine phosphatase isoform X2 [Polistes dominula];XP_015171751.1 PREDICTED: phosphoserine phosphatase isoform X1 [Polistes dominula]	2	2	2	2	25,17	0	5435800	0	0
XP_015171804.1;XP_015171803.1;XP_015171802.1;XP_015171801.1	XP_015171804.1;XP_015171803.1;XP_015171802.1;XP_015171801.1	XP_015171804.1 PREDICTED: renin receptor isoform X4 [Polistes dominula];XP_015171803.1 PREDICTED: renin receptor isoform X3 [Polistes dominula];XP_015171802.1 PREDICTED: renin receptor isoform X2 [Polistes dominula];XP_015171801.1 PREDICTED: renin receptor	4	2	2	2	47,549	8041900	0	0	0
XP_015171815.1	XP_015171815.1	XP_015171815.1 PREDICTED: peroxiredoxin-6-like [Polistes dominula]	1	2	2	2	24,971	12336000	0	0	0
XP_015172205.1	XP_015172205.1	XP_015172205.1 PREDICTED: chloride intracellular channel exc-4 [Polistes dominula]	1	2	2	2	29,846	0	0	0	0
XP_015172559.1;XP_015172558.1	XP_015172559.1;XP_015172558.1	XP_015172559.1 PREDICTED: poly(U)-specific endoribonuclease homolog [Polistes dominula];XP_015172558.1 PREDICTED: poly(U)-specific endoribonuclease homolog [Polistes dominula]	2	2	2	2	37,791	2040700	6230400	0	0
XP_015172580.1	XP_015172580.1	XP_015172580.1 PREDICTED: plasminogen activator inhibitor 1 RNA-binding protein [Polistes dominula]	1	2	2	2	47,78	0	0	0	0
XP_015188342.1;XP_015172763.1	XP_015188342.1;XP_015172763.1	XP_015188342.1 PREDICTED: 40S ribosomal protein S28-like [Polistes dominula];XP_015172763.1 PREDICTED: 40S ribosomal protein S28 [Polistes dominula]	2	2	2	2	7,3996	11708000	4247600	20585000	0
XP_015181269.1;XP_015177524.1;XP_015173448.1	XP_015181269.1;XP_015177524.1;XP_015173448.1	XP_015181269.1 PREDICTED: histone H2B [Polistes dominula];XP_015177524.1 PREDICTED: histone H2B-like [Polistes dominula];XP_015173448.1 PREDICTED: histone H2B-like [Polistes dominula]	3	2	2	2	13,728	20928000	0	0	0

XP_015173468.1;XP_015173467.1;XP_015173466.1;XP_015173465.1;XP_015173464.1;XP_015173463.1;XP_015173461.1;XP_015173460.1	XP_015173468.1;XP_015173467.1;XP_015173466.1;XP_015173465.1;XP_015173464.1;XP_015173463.1;XP_015173461.1;XP_015173460.1	XP_015173468.1 PREDICTED: uncharacterized protein LOC107064856 isoform X6 [Polistes dominula];XP_015173467.1 PREDICTED: uncharacterized protein LOC107064856 isoform X5 [Polistes dominula];XP_015173466.1 PREDICTED: uncharacterized protein LOC107064856 isofo	8	2	2	2	100,95	0	0	0	1002900
XP_015173510.1	XP_015173510.1	XP_015173510.1 PREDICTED: sorting nexin-12 [Polistes dominula]	1	2	2	2	19,045	0	0	0	0
XP_015174063.1;XP_015174062.1;XP_015174061.1;XP_015174060.1	XP_015174063.1;XP_015174062.1;XP_015174061.1;XP_015174060.1	XP_015174063.1 PREDICTED: alpha-tocopherol transfer protein-like isoform X2 [Polistes dominula];XP_015174062.1 PREDICTED: alpha-tocopherol transfer protein-like isoform X2 [Polistes dominula];XP_015174061.1 PREDICTED: alpha-tocopherol transfer protein-like	4	2	2	2	35,19	5004800	0	0	0
XP_015174070.1;XP_015174069.1;XP_015174068.1;XP_015174072.1;XP_015174071.1;XP_015174067.1;XP_015174066.1;XP_015174065.1;XP_015174064.1	XP_015174070.1;XP_015174069.1;XP_015174068.1;XP_015174072.1;XP_015174071.1;XP_015174067.1;XP_015174066.1;XP_015174065.1;XP_015174064.1	XP_015174070.1 PREDICTED: vinculin isoform X7 [Polistes dominula];XP_015174069.1 PREDICTED: vinculin isoform X6 [Polistes dominula];XP_015174068.1 PREDICTED: vinculin isoform X5 [Polistes dominula];XP_015174072.1 PREDICTED: vinculin isoform X9 [Polistes do	9	2	2	2	107,16	1088100	0	0	0
XP_015174105.1	XP_015174105.1	XP_015174105.1 PREDICTED: glycine-rich cell wall structural protein-like [Polistes dominula]	1	2	2	2	39,436	0	84925000	0	0
XP_015174139.1	XP_015174139.1	XP_015174139.1 PREDICTED: mesencephalic astrocyte-derived neurotrophic factor homolog [Polistes dominula]	1	2	2	2	20,189	0	21328000	7259700	2953000
XP_015174186.1	XP_015174186.1	XP_015174186.1 PREDICTED: aldose 1-epimerase [Polistes dominula]	1	2	2	2	41,641	0	58296000	0	0
XP_015174359.1;XP_015174358.1;XP_015174356.1;XP_015174357.1	XP_015174359.1;XP_015174358.1;XP_015174356.1;XP_015174357.1	XP_015174359.1 PREDICTED: fasciclin-2 isoform X4 [Polistes dominula];XP_015174358.1 PREDICTED: fasciclin-2 isoform X3 [Polistes dominula];XP_015174356.1 PREDICTED: fasciclin-2 isoform X1 [Polistes dominula];XP_015174357.1 PREDICTED: fasciclin-2 isoform X2	4	2	2	2	88,348	4441700	0	0	0
XP_015174371.1;XP_015174370.1	XP_015174371.1;XP_015174370.1	XP_015174371.1 PREDICTED: leukocyte elastase inhibitor-like isoform X2 [Polistes dominula];XP_015174370.1 PREDICTED: leukocyte elastase inhibitor-like isoform X1 [Polistes dominula]	2	2	2	2	49,615	0	34111000	0	0
XP_015174516.1;XP_015174515.1	XP_015174516.1;XP_015174515.1	XP_015174516.1 PREDICTED: peptidyl-prolyl cis-trans isomerase FKBP4-like isoform X2 [Polistes dominula];XP_015174515.1 PREDICTED: FK506-binding protein 59-like isoform X1 [Polistes dominula]	2	2	2	2	45,63	0	25483000	0	0

XP_015174856.1	XP_015174856.1	XP_015174856.1 PREDICTED: serine/threonine-protein phosphatase 2A activator-like [Polistes dominula]	1	2	2	2	38,447	0	8750300	0	0
XP_015175066.1	XP_015175066.1	XP_015175066.1 PREDICTED: magnesium-dependent phosphatase 1-like [Polistes dominula]	1	2	2	2	18,845	0	10166000	0	0
XP_015175125.1	XP_015175125.1	XP_015175125.1 PREDICTED: estradiol 17-beta-dehydrogenase 8-like [Polistes dominula]	1	2	2	2	26,658	0	9737600	0	0
XP_015175375.1;XP_015175374.1	XP_015175375.1;XP_015175374.1	XP_015175375.1 PREDICTED: 40S ribosomal protein S12 isoform X2 [Polistes dominula];XP_015175374.1 PREDICTED: 40S ribosomal protein S12 isoform X1 [Polistes dominula]	2	2	2	2	13,541	0	19166000	0	0
XP_015175542.1	XP_015175542.1	XP_015175542.1 PREDICTED: transitional endoplasmic reticulum ATPase TER94 [Polistes dominula]	1	2	2	2	88,693	2806900	0	0	0
XP_015175593.1	XP_015175593.1	XP_015175593.1 PREDICTED: uncharacterized protein LOC107065962 [Polistes dominula]	1	2	2	2	33,21	0	0	0	0
XP_015175623.1;XP_015187333.1;XP_015187332.1	XP_015175623.1;XP_015187333.1;XP_015187332.1	XP_015175623.1 PREDICTED: acetylcholinesterase [Polistes dominula];XP_015187333.1 PREDICTED: venom carboxylesterase-6-like isoform X2 [Polistes dominula];XP_015187332.1 PREDICTED: esterase FE4-like isoform X1 [Polistes dominula]	3	2	2	2	62,512	0	7388200	0	0
XP_015175776.1;XP_015175775.1	XP_015175776.1;XP_015175775.1	XP_015175776.1 PREDICTED: regulator of chromosome condensation-like isoform X2 [Polistes dominula];XP_015175775.1 PREDICTED: regulator of chromosome condensation-like isoform X1 [Polistes dominula]	2	2	2	2	46,03	0	0	0	0
XP_015175901.1;XP_015175900.1;XP_015175899.1	XP_015175901.1;XP_015175900.1;XP_015175899.1	XP_015175901.1 PREDICTED: protein enhancer of sevenless 2B [Polistes dominula];XP_015175900.1 PREDICTED: protein enhancer of sevenless 2B [Polistes dominula];XP_015175899.1 PREDICTED: protein enhancer of sevenless 2B [Polistes dominula]	3	2	2	2	24,746	0	7903600	7532900	2394600
XP_015176317.1	XP_015176317.1	XP_015176317.1 PREDICTED: peptidyl-prolyl cis-trans isomerase FKBP1A [Polistes dominula]	1	2	2	2	11,86	7136400	0	67439000	3240300
XP_015176386.1	XP_015176386.1	XP_015176386.1 PREDICTED: abnormal spindle-like microcephaly-associated protein homolog [Polistes dominula]	1	2	2	2	15,539	0	0	0	0
XP_015176486.1	XP_015176486.1	XP_015176486.1 PREDICTED: ubiquitin-like protein 5 [Polistes dominula]	1	2	2	2	8,681	0	0	0	0
XP_015176527.1	XP_015176527.1	XP_015176527.1 PREDICTED: lysozyme c-1-like [Polistes dominula]	1	2	2	2	18,23	33063000	0	0	0
XP_015176929.1	XP_015176929.1	XP_015176929.1 PREDICTED: proline dehydrogenase 1, mitochondrial [Polistes dominula]	1	2	2	2	71,853	6400500	0	0	0
XP_015176930.1	XP_015176930.1	XP_015176930.1 PREDICTED: profilin [Polistes dominula]	1	2	2	2	13,715	8910100	0	0	0
XP_015177131.1;XP_015189964.1;XP_015189970.1	XP_015177131.1;XP_015189964.1;XP_015189970.1	XP_015177131.1 PREDICTED: titin [Polistes dominula];XP_015189964.1 PREDICTED: RNA-binding protein 12B-like, partial [Polistes dominula];XP_015189970.1 PREDICTED: RNA-binding protein 12B-like, partial [Polistes dominula]	3	2	2	2	438,01	0	0	0	0

XP_015177284.1;XP_015177283.1;XP_015177282.1;XP_015177281.1;XP_015177280.1;XP_015177279.1	XP_015177284.1;XP_015177283.1;XP_015177282.1;XP_015177281.1;XP_015177280.1;XP_015177279.1	XP_015177284.1 PREDICTED: coronin-6 isoform X3 [Polistes dominula];XP_015177283.1 PREDICTED: coronin-6 isoform X3 [Polistes dominula];XP_015177282.1 PREDICTED: coronin-6 isoform X3 [Polistes dominula];XP_015177281.1 PREDICTED: coronin-6 isoform X2 [Poliste	6	2	2	2	54,775	0	3853600	0	842190
XP_015177311.1	XP_015177311.1	XP_015177311.1 PREDICTED: ubiquitin carboxyl-terminal hydrolase 14 [Polistes dominula]	1	2	2	2	56,986	0	0	0	0
XP_015177806.1	XP_015177806.1	XP_015177806.1 PREDICTED: ubiquitin-conjugating enzyme E2-17 kDa [Polistes dominula]	1	2	2	2	16,678	0	35033000	0	0
XP_015177856.1	XP_015177856.1	XP_015177856.1 PREDICTED: uroporphyrinogen decarboxylase [Polistes dominula]	1	2	2	2	39,809	0	10567000	0	0
XP_015178047.1;XP_015178045.1	XP_015178047.1;XP_015178045.1	XP_015178047.1 PREDICTED: SUMO-conjugating enzyme UBC9-B [Polistes dominula];XP_015178045.1 PREDICTED: SUMO-conjugating enzyme UBC9-B [Polistes dominula]	2	2	2	2	18,135	0	24148000	0	0
XP_015178129.1;XP_015178128.1;XP_015178127.1	XP_015178129.1;XP_015178128.1;XP_015178127.1	XP_015178129.1 PREDICTED: beta-mannosidase isoform X3 [Polistes dominula];XP_015178128.1 PREDICTED: beta-mannosidase isoform X2 [Polistes dominula];XP_015178127.1 PREDICTED: beta-mannosidase isoform X1 [Polistes dominula]	3	2	2	2	89,441	0	8460400	0	0
XP_015178184.1	XP_015178184.1	XP_015178184.1 PREDICTED: adipocyte plasma membrane-associated protein-like [Polistes dominula]	1	2	2	2	75,446	0	6701700	0	0
XP_015178221.1;XP_015178220.1;XP_015178219.1	XP_015178221.1;XP_015178220.1;XP_015178219.1	XP_015178221.1 PREDICTED: aspartate--tRNA ligase, cytoplasmic [Polistes dominula];XP_015178220.1 PREDICTED: aspartate--tRNA ligase, cytoplasmic [Polistes dominula];XP_015178219.1 PREDICTED: aspartate--tRNA ligase, cytoplasmic [Polistes dominula]	3	2	2	2	60,022	0	10686000	0	0
XP_015178376.1	XP_015178376.1	XP_015178376.1 PREDICTED: PC4 and SFRS1-interacting protein [Polistes dominula]	1	2	2	2	63,542	0	0	0	32234000
XP_015178653.1	XP_015178653.1	XP_015178653.1 PREDICTED: ubiquitin domain-containing protein UBFD1-like [Polistes dominula]	1	2	2	2	34,426	0	7018000	0	0
XP_015178743.1	XP_015178743.1	XP_015178743.1 PREDICTED: uncharacterized protein LOC107067604 [Polistes dominula]	1	2	2	2	342,16	0	0	0	0
XP_015178779.1	XP_015178779.1	XP_015178779.1 PREDICTED: deoxyribose-phosphate aldolase [Polistes dominula]	1	2	2	2	34,508	0	5537900	17133000	0
XP_015178800.1	XP_015178800.1	XP_015178800.1 PREDICTED: LDLR chaperone boca [Polistes dominula]	1	2	2	2	23,378	0	18221000	0	0
XP_015178833.1;XP_015178832.1;XP_015178831.1;XP_015178830.1;XP_015178829.1;XP_015178828.1	XP_015178833.1;XP_015178832.1;XP_015178831.1;XP_015178830.1;XP_015178829.1;XP_015178828.1	XP_015178833.1 PREDICTED: 2-oxoglutarate dehydrogenase, mitochondrial isoform X6 [Polistes dominula];XP_015178832.1 PREDICTED: 2-oxoglutarate dehydrogenase, mitochondrial isoform X5 [Polistes dominula];XP_015178831.1 PREDICTED: 2-oxoglutarate dehydrogenase	6	2	2	2	115,32	2708900	0	0	0
XP_015178967.1	XP_015178967.1	XP_015178967.1 PREDICTED: ATP synthase subunit O, mitochondrial [Polistes dominula]	1	2	2	2	22,576	15805000	0	0	0

XP_015179039.1	XP_015179039.1	XP_015179039.1 PREDICTED: coiled-coil domain-containing protein 58 [Polistes dominula]	1	2	2	2	16,738	0	4936300	0	0
XP_015179070.1	XP_015179070.1	XP_015179070.1 PREDICTED: la protein homolog [Polistes dominula]	1	2	2	2	51,819	0	0	795920000	0
XP_015179217.1;XP_015179216.1;XP_015179215.1;XP_015179214.1;XP_015179213.1;XP_015179211.1;XP_015179218.1	XP_015179217.1;XP_015179216.1;XP_015179215.1;XP_015179214.1;XP_015179213.1;XP_015179211.1;XP_015179218.1	XP_015179217.1 PREDICTED: RNA-binding protein 4.1-like isoform X5 [Polistes dominula];XP_015179216.1 PREDICTED: RNA-binding protein 4.1-like isoform X4 [Polistes dominula];XP_015179215.1 PREDICTED: RNA-binding protein 4-like isoform X3 [Polistes dominula];	7	2	2	2	35,483	0	0	0	0
XP_015179260.1	XP_015179260.1	XP_015179260.1 PREDICTED: protein NPC2 homolog [Polistes dominula]	1	2	2	2	16,763	0	23675000	0	23750000
XP_015179768.1	XP_015179768.1	XP_015179768.1 PREDICTED: translation machinery-associated protein 7 homolog [Polistes dominula]	1	2	2	2	6,937	0	131250000	0	0
XP_015179786.1	XP_015179786.1	XP_015179786.1 PREDICTED: protein CDV3 homolog [Polistes dominula]	1	2	2	2	27,699	0	0	0	0
XP_015180233.1	XP_015180233.1	XP_015180233.1 PREDICTED: very long-chain specific acyl-CoA dehydrogenase, mitochondrial [Polistes dominula]	1	2	2	2	69,648	17916000	0	0	0
XP_015180247.1	XP_015180247.1	XP_015180247.1 PREDICTED: venom acid phosphatase Acph-1-like [Polistes dominula]	1	2	2	2	47,808	0	11156000	0	0
XP_015180359.1	XP_015180359.1	XP_015180359.1 PREDICTED: probable aspartate aminotransferase, cytoplasmic [Polistes dominula]	1	2	2	2	46,631	6473300	0	0	2048800
XP_015180559.1	XP_015180559.1	XP_015180559.1 PREDICTED: uncharacterized protein LOC107068554 [Polistes dominula]	1	2	2	2	181,35	0	23832000	0	26839000
XP_015180879.1	XP_015180879.1	XP_015180879.1 PREDICTED: mitochondrial fission 1 protein-like isoform X2 [Polistes dominula]	1	2	2	2	16,924	4624000	7045600	5393200	0
XP_015181119.1;XP_015181118.1	XP_015181119.1;XP_015181118.1	XP_015181119.1 PREDICTED: syntaxin-7-like [Polistes dominula];XP_015181118.1 PREDICTED: syntaxin-7-like [Polistes dominula]	2	2	2	2	46,093	0	2614900	0	0
XP_015181383.1	XP_015181383.1	XP_015181383.1 PREDICTED: calcyclin-binding protein [Polistes dominula]	1	2	2	2	25,939	0	0	0	0
XP_015181480.1;XP_015181479.1;XP_015181478.1	XP_015181480.1;XP_015181479.1;XP_015181478.1	XP_015181480.1 PREDICTED: transcription factor BTF3 homolog 4 [Polistes dominula];XP_015181479.1 PREDICTED: transcription factor BTF3 homolog 4 [Polistes dominula];XP_015181478.1 PREDICTED: transcription factor BTF3 homolog 4 [Polistes dominula]	3	2	2	2	20,067	0	0	0	0
XP_015181547.1	XP_015181547.1	XP_015181547.1 PREDICTED: kielin/chordin-like protein [Polistes dominula]	1	2	2	2	35,413	11615000	0	0	0
XP_015181571.1;XP_015181570.1;XP_015181569.1;XP_015181568.1;XP_015181567.1	XP_015181571.1;XP_015181570.1;XP_015181569.1;XP_015181568.1;XP_015181567.1	XP_015181571.1 PREDICTED: putative uncharacterized protein DDB_G0290521 isoform X4 [Polistes dominula];XP_015181570.1 PREDICTED: putative uncharacterized protein DDB_G0290521 isoform X3 [Polistes dominula];XP_015181569.1 PREDICTED: extensin-like isoform X2	5	2	2	2	30,676	0	0	143180000	0

XP_015181736.1	XP_015181736.1	XP_015181736.1 PREDICTED: methionine aminopeptidase 1 [Polistes dominula]	1	2	2	2	42,308	0	0	0	0
XP_015181824.1	XP_015181824.1	XP_015181824.1 PREDICTED: dehydrogenase/reductase SDR family member 11-like [Polistes dominula]	1	2	2	2	26,94	0	8687300	0	0
XP_015181987.1	XP_015181987.1	XP_015181987.1 PREDICTED: GMP reductase 1-like [Polistes dominula]	1	2	2	2	37,86	0	2866000	0	1185100
XP_015182048.1	XP_015182048.1	XP_015182048.1 PREDICTED: histidine triad nucleotide-binding protein 1 [Polistes dominula]	1	2	2	2	17,31	0	15030000	0	3111900
XP_015182363.1;XP_015182362.1	XP_015182363.1;XP_015182362.1	XP_015182363.1 PREDICTED: extended synaptotagmin-2 isoform X2 [Polistes dominula];XP_015182362.1 PREDICTED: extended synaptotagmin-2 isoform X1 [Polistes dominula]	2	2	2	2	90,538	0	0	0	0
XP_015182425.1	XP_015182425.1	XP_015182425.1 PREDICTED: isopentenyl-diphosphate Delta-isomerase 1 [Polistes dominula]	1	2	2	2	28,583	0	3417300	0	0
XP_015182548.1	XP_015182548.1	XP_015182548.1 PREDICTED: uncharacterized protein LOC107069612 [Polistes dominula]	1	2	2	2	9,6082	0	37417000	0	0
XP_015182894.1	XP_015182894.1	XP_015182894.1 PREDICTED: 40S ribosomal protein S10-like [Polistes dominula]	1	2	2	2	18,008	3360400	0	0	0
XP_015183173.1	XP_015183173.1	XP_015183173.1 PREDICTED: coiled-coil domain-containing protein 25 [Polistes dominula]	1	2	2	2	24,592	0	3650400	0	0
XP_015183299.1	XP_015183299.1	XP_015183299.1 PREDICTED: Na(+)/H(+) exchange regulatory cofactor NHE-RF1 [Polistes dominula]	1	2	2	2	30,333	23566000	0	0	2695300
XP_015183432.1;XP_015183431.1;XP_015183430.1;XP_015183429.1;XP_015183428.1;XP_015183427.1	XP_015183432.1;XP_015183431.1;XP_015183430.1;XP_015183429.1;XP_015183428.1;XP_015183427.1	XP_015183432.1 PREDICTED: protein tyrosine phosphatase type IVA 1 [Polistes dominula];XP_015183431.1 PREDICTED: protein tyrosine phosphatase type IVA 1 [Polistes dominula];XP_015183430.1 PREDICTED: protein tyrosine phosphatase type IVA 1 [Polistes dominula	6	2	2	2	20,109	0	0	0	0
XP_015184603.1;XP_015184602.1;XP_015184600.1;XP_015184599.1	XP_015184603.1;XP_015184602.1;XP_015184600.1;XP_015184599.1	XP_015184603.1 PREDICTED: aminopeptidase N isoform X2 [Polistes dominula];XP_015184602.1 PREDICTED: aminopeptidase N isoform X2 [Polistes dominula];XP_015184600.1 PREDICTED: aminopeptidase N isoform X2 [Polistes dominula];XP_015184599.1 PREDICTED: aminopep	4	2	2	2	113,23	3093800	2240000	0	0
XP_015184717.1;XP_015184716.1	XP_015184717.1;XP_015184716.1	XP_015184717.1 PREDICTED: uncharacterized protein DDB_G0286299-like [Polistes dominula];XP_015184716.1 PREDICTED: uncharacterized protein DDB_G0286299-like [Polistes dominula]	2	2	2	2	42,272	0	0	0	0
XP_015185027.1;XP_015185026.1;XP_015185024.1;XP_015185023.1	XP_015185027.1;XP_015185026.1;XP_015185024.1;XP_015185023.1	XP_015185027.1 PREDICTED: peroxiredoxin-5, mitochondrial isoform X2 [Polistes dominula];XP_015185026.1 PREDICTED: peroxiredoxin-5, mitochondrial isoform X2 [Polistes dominula];XP_015185024.1 PREDICTED: peroxiredoxin-5, mitochondrial isoform X2 [Polistes do	4	2	2	2	16,732	36176000	0	0	0
XP_015185108.1	XP_015185108.1	XP_015185108.1 PREDICTED: pyrimidodiazepine synthase-like [Polistes dominula]	1	2	2	2	27,772	0	10368000	0	0
XP_015185205.1	XP_015185205.1	XP_015185205.1 PREDICTED: protein FAM32A [Polistes dominula]	1	2	2	2	13,712	0	14181000000	2184200000	714470000

XP_015185277.1;XP_015185276.1	XP_015185277.1;XP_015185276.1	XP_015185277.1 PREDICTED: F-actin-capping protein subunit beta isoform X2 [Polistes dominula];XP_015185276.1 PREDICTED: F-actin-capping protein subunit beta isoform X1 [Polistes dominula]	2	2	2	2	30,203	0	0	22469000	0
XP_015185385.1	XP_015185385.1	XP_015185385.1 PREDICTED: ribosome-recycling factor, mitochondrial [Polistes dominula]	1	2	2	2	33,38	0	0	0	0
XP_015185661.1;XP_015185660.1;XP_015185659.1;XP_015185658.1;XP_015185657.1;XP_015185656.1;XP_015185655.1;XP_015185654.1;XP_015185653.1	XP_015185661.1;XP_015185660.1;XP_015185659.1;XP_015185658.1;XP_015185657.1;XP_015185656.1;XP_015185655.1;XP_015185654.1;XP_015185653.1	XP_015185661.1 PREDICTED: ras-related protein Rab-5B [Polistes dominula];XP_015185660.1 PREDICTED: ras-related protein Rab-5B [Polistes dominula];XP_015185659.1 PREDICTED: ras-related protein Rab-5B [Polistes dominula];XP_015185658.1 PREDICTED: ras-related	9	2	2	2	23,472	0	0	0	0
XP_015185668.1;XP_015185666.1	XP_015185668.1;XP_015185666.1	XP_015185668.1 PREDICTED: UTP--glucose-1-phosphate uridylyltransferase isoform X2 [Polistes dominula];XP_015185666.1 PREDICTED: UTP--glucose-1-phosphate uridylyltransferase isoform X1 [Polistes dominula]	2	2	2	2	57,566	8563900	0	0	0
XP_015185678.1	XP_015185678.1	XP_015185678.1 PREDICTED: DNA ligase 1-like [Polistes dominula]	1	2	2	2	38,872	0	0	0	0
XP_015185699.1;XP_015185698.1	XP_015185699.1;XP_015185698.1	XP_015185699.1 PREDICTED: uncharacterized protein LOC107071317 isoform X2 [Polistes dominula];XP_015185698.1 PREDICTED: uncharacterized protein LOC107071317 isoform X1 [Polistes dominula]	2	2	2	2	12,343	0	4360200	0	0
XP_015185741.1	XP_015185741.1	XP_015185741.1 PREDICTED: eukaryotic initiation factor 4A-I [Polistes dominula]	1	2	2	2	48,227	7210700	0	0	0
XP_015186195.1;XP_015186194.1	XP_015186195.1;XP_015186194.1	XP_015186195.1 PREDICTED: regucalcin-like isoform X2 [Polistes dominula];XP_015186194.1 PREDICTED: regucalcin-like isoform X1 [Polistes dominula]	2	2	2	2	34,971	0	21340000	14752000	4361000
XP_015186274.1;XP_015186273.1	XP_015186274.1;XP_015186273.1	XP_015186274.1 PREDICTED: vigilin [Polistes dominula];XP_015186273.1 PREDICTED: vigilin [Polistes dominula]	2	2	2	2	139,5	0	0	0	0
XP_015186648.1;XP_015186647.1	XP_015186648.1;XP_015186647.1	XP_015186648.1 PREDICTED: uncharacterized protein LOC107071834 isoform X2 [Polistes dominula];XP_015186647.1 PREDICTED: uncharacterized protein LOC107071834 isoform X1 [Polistes dominula]	2	2	2	2	49,189	12998000	0	0	0

XP_015186747.1;XP_015186749.1;XP_015186745.1;XP_015186744.1;XP_015186743.1;XP_015186748.1;XP_015186742.1;XP_015186741.1;XP_015186740.1;XP_015186739.1;XP_015186738.1	XP_015186747.1;XP_015186749.1;XP_015186745.1;XP_015186744.1;XP_015186743.1;XP_015186748.1;XP_015186742.1;XP_015186741.1;XP_015186740.1;XP_015186739.1;XP_015186738.1	XP_015186747.1 PREDICTED: myosin heavy chain, muscle isoform X9 [Polistes dominula];XP_015186749.1 PREDICTED: myosin heavy chain, muscle isoform X11 [Polistes dominula];XP_015186745.1 PREDICTED: myosin heavy chain, muscle isoform X8 [Polistes dominula];XP_	11	2	2	2	223,25	0	29084000	0	0
XP_015187200.1	XP_015187200.1	XP_015187200.1 PREDICTED: transcriptional adapter 2B isoform X13 [Polistes dominula]	1	2	2	2	69,471	2026400	64608000	52067000	14478000
XP_015187475.1	XP_015187475.1	XP_015187475.1 PREDICTED: partner of Y14 and mago [Polistes dominula]	1	2	2	2	23,633	0	0	0	0
XP_015188110.1;XP_015188108.1;XP_015188107.1;XP_015188109.1	XP_015188110.1;XP_015188108.1;XP_015188107.1;XP_015188109.1	XP_015188110.1 PREDICTED: dystroglycan-like isoform X3 [Polistes dominula];XP_015188108.1 PREDICTED: dystroglycan-like isoform X1 [Polistes dominula];XP_015188107.1 PREDICTED: dystroglycan-like isoform X1 [Polistes dominula];XP_015188109.1 PREDICTED: dystr	4	2	2	2	130,1	4315800	0	0	0
XP_015188170.1	XP_015188170.1	XP_015188170.1 PREDICTED: T-complex protein 1 subunit delta [Polistes dominula]	1	2	2	2	57,302	3201800	0	0	0
XP_015188273.1	XP_015188273.1	XP_015188273.1 PREDICTED: actin-interacting protein 1 [Polistes dominula]	1	2	2	2	66,201	0	0	0	7357300
XP_015188465.1;XP_015188456.1	XP_015188465.1;XP_015188456.1	XP_015188465.1 PREDICTED: lamin Dm0-like isoform X2 [Polistes dominula];XP_015188456.1 PREDICTED: lamin Dm0-like isoform X1 [Polistes dominula]	2	2	2	2	69,636	0	0	0	3203000
XP_015188476.1	XP_015188476.1	XP_015188476.1 PREDICTED: protein SCO1 homolog, mitochondrial [Polistes dominula]	1	2	2	2	29,851	0	4410900	0	0
XP_015188938.1	XP_015188938.1	XP_015188938.1 PREDICTED: uncharacterized protein LOC107073047 [Polistes dominula]	1	2	2	2	27,7	0	0	0	0
XP_015189095.1;XP_015189094.1	XP_015189095.1;XP_015189094.1	XP_015189095.1 PREDICTED: WAP four-disulfide core domain protein 3-like [Polistes dominula];XP_015189094.1 PREDICTED: WAP four-disulfide core domain protein 3-like [Polistes dominula]	2	2	2	2	15,443	30451000	0	0	0

XP_015189606.1;XP_015189605.1;XP_015189587.1;XP_015189588.1;XP_015189589.1;XP_015189590.1;XP_015189591.1;XP_015189592.1;XP_015189593.1;XP_015189594.1;XP_015189595.1;XP_015189596.1;XP_015189597.1;XP_015189598.1;XP_015189600.1;XP_015189602.1;XP_015189603.1;XP_015189604.1;XP_015189586.1;XP_015189599.1	XP_015189606.1;XP_015189605.1;XP_015189587.1;XP_015189588.1;XP_015189589.1;XP_015189590.1;XP_015189591.1;XP_015189592.1;XP_015189593.1;XP_015189594.1;XP_015189595.1;XP_015189596.1;XP_015189597.1;XP_015189598.1;XP_015189600.1;XP_015189602.1;XP_015189603.1;XP_015189604.1;XP_015189586.1;XP_015189599.1	XP_015189606.1 PREDICTED: phosphatidylinositol-binding clathrin assembly protein LAP isoform X16 [Polistes dominula];XP_015189605.1 PREDICTED: phosphatidylinositol-binding clathrin assembly protein LAP isoform X15 [Polistes dominula];XP_015189587.1 PREDICT	20	2	2	2	83,198	0	2761400	0	0
XP_015189995.1	XP_015189995.1	XP_015189995.1 PREDICTED: ras-related protein Rab-2 [Polistes dominula]	1	2	2	2	23,663	10984000	0	0	0
XP_015190028.1;XP_015190027.1;XP_015190026.1;XP_015190024.1;XP_015190023.1	XP_015190028.1;XP_015190027.1;XP_015190026.1;XP_015190024.1;XP_015190023.1	XP_015190028.1 PREDICTED: myosin-11 isoform X5 [Polistes dominula];XP_015190027.1 PREDICTED: myosin-11 isoform X4 [Polistes dominula];XP_015190026.1 PREDICTED: myosin-11 isoform X3 [Polistes dominula];XP_015190024.1 PREDICTED: myosin-11 isoform X2 [Poliste	5	2	2	2	156,49	0	0	19380000	0
XP_015190318.1	XP_015190318.1	XP_015190318.1 PREDICTED: phosphoglucomutase-2 [Polistes dominula]	1	2	2	2	68,448	0	5985600	0	0
XP_015190431.1	XP_015190431.1	XP_015190431.1 PREDICTED: protein amalgam-like [Polistes dominula]	1	2	2	2	57,664	8815800	0	0	0
XP_015190708.1	XP_015190708.1	XP_015190708.1 PREDICTED: myosin regulatory light chain 2 [Polistes dominula]	1	2	2	2	23,277	0	968690000	232630000	363830000
XP_015190984.1	XP_015190984.1	XP_015190984.1 PREDICTED: H/ACA ribonucleoprotein complex subunit 4 [Polistes dominula]	1	2	2	2	61,077	0	2228600	0	0
XP_015191283.1	XP_015191283.1	XP_015191283.1 PREDICTED: selenide, water dikinase [Polistes dominula]	1	2	2	2	45,101	4126000	13964000	0	0
XP_015191327.1	XP_015191327.1	XP_015191327.1 PREDICTED: claspin-like [Polistes dominula]	1	2	2	2	38,061	0	7900600	0	0

XP_015191348.1	XP_015191348.1	XP_015191348.1 PREDICTED: inositol monophosphatase 2-like [Polistes dominula]	1	2	2	2	31,119	0	0	4804700	0
XP_015191423.1;XP_015191422.1;XP_015191421.1;XP_015191420.1;XP_015191424.1	XP_015191423.1;XP_015191422.1;XP_015191421.1;XP_015191420.1;XP_015191424.1	XP_015191423.1 PREDICTED: alpha-actinin, sarcomeric isoform X2 [Polistes dominula];XP_015191422.1 PREDICTED: alpha-actinin, sarcomeric isoform X1 [Polistes dominula];XP_015191421.1 PREDICTED: alpha-actinin, sarcomeric isoform X1 [Polistes dominula];XP_0151	5	2	2	2	103,11	0	0	0	6195000
XP_015191586.1;XP_015191585.1	XP_015191586.1;XP_015191585.1	XP_015191586.1 PREDICTED: activated RNA polymerase II transcriptional coactivator p15 isoform X2 [Polistes dominula];XP_015191585.1 PREDICTED: activated RNA polymerase II transcriptional coactivator p15 isoform X1 [Polistes dominula]	2	2	2	2	13,775	0	0	0	0
XP_015171239.1;XP_015171238.1;XP_015171237.1;XP_015171236.1;XP_015171235.1	XP_015171239.1;XP_015171238.1;XP_015171237.1;XP_015171236.1;XP_015171235.1	XP_015171239.1 PREDICTED: RNA-binding protein Nova-1 isoform X4 [Polistes dominula];XP_015171238.1 PREDICTED: RNA-binding protein Nova-1 isoform X3 [Polistes dominula];XP_015171237.1 PREDICTED: RNA-binding protein Nova-1 isoform X2 [Polistes dominula];XP_0	5	3	3	3	43,379	0	0	0	0
XP_015172226.1;XP_015172225.1	XP_015172226.1;XP_015172225.1	XP_015172226.1 PREDICTED: succinate-semialdehyde dehydrogenase [NADP(+)] GabD [Polistes dominula];XP_015172225.1 PREDICTED: succinate-semialdehyde dehydrogenase [NADP(+)] GabD [Polistes dominula]	2	3	3	3	54,63	0	0	0	0
XP_015179060.1;XP_015184748.1;XP_015172916.1;XP_015172915.1;XP_015172655.1;XP_015186969.1	XP_015179060.1;XP_015184748.1;XP_015172916.1;XP_015172915.1;XP_015172655.1;XP_015186969.1	XP_015179060.1 PREDICTED: ubiquitin [Polistes dominula];XP_015184748.1 PREDICTED: ubiquitin-60S ribosomal protein L40 [Polistes dominula];XP_015172916.1 PREDICTED: ubiquitin-40S ribosomal protein S27a [Polistes dominula];XP_015172915.1 PREDICTED: ubiquitin	6	3	3	3	12,845	0	134630000	17329000	52528000
XP_015173290.1	XP_015173290.1	XP_015173290.1 PREDICTED: omega-amidase NIT2 [Polistes dominula]	1	3	3	3	34,148	0	58685000	0	0
XP_015173359.1	XP_015173359.1	XP_015173359.1 PREDICTED: 4-coumarate--CoA ligase 1-like [Polistes dominula]	1	3	3	3	53,898	4215500	13845000	0	0
XP_015173532.1	XP_015173532.1	XP_015173532.1 PREDICTED: V-type proton ATPase subunit G [Polistes dominula]	1	3	3	3	13,716	18065000	36783000	11315000	0
XP_015173963.1	XP_015173963.1	XP_015173963.1 PREDICTED: trifunctional enzyme subunit alpha, mitochondrial [Polistes dominula]	1	3	3	3	82,364	0	0	0	0
XP_015174089.1	XP_015174089.1	XP_015174089.1 PREDICTED: ubiquitin-conjugating enzyme E2 variant 2 [Polistes dominula]	1	3	3	3	16,334	0	0	0	0
XP_015174199.1;XP_015174196.1	XP_015174199.1;XP_015174196.1	XP_015174199.1 PREDICTED: heterogeneous nuclear ribonucleoprotein R isoform X5 [Polistes dominula];XP_015174196.1 PREDICTED: heterogeneous nuclear ribonucleoprotein R isoform X2 [Polistes dominula]	2	3	3	2	74,425	0	14973000	0	1875700
XP_015174225.1	XP_015174225.1	XP_015174225.1 PREDICTED: probable medium-chain specific acyl-CoA dehydrogenase, mitochondrial [Polistes dominula]	1	3	3	3	46,395	36979000	1934500	0	0

XP_015174579.1;XP_015174578.1	XP_015174579.1;XP_015174578.1	XP_015174579.1 PREDICTED: putative serine protease K12H4.7 [Polistes dominula];XP_015174578.1 PREDICTED: putative serine protease K12H4.7 [Polistes dominula]	2	3	3	3	56,184	0	9016900	0	0
XP_015174636.1	XP_015174636.1	XP_015174636.1 PREDICTED: lachesin [Polistes dominula]	1	3	3	3	40,528	11325000	10343000	0	0
XP_015174663.1;XP_015174662.1	XP_015174663.1;XP_015174662.1	XP_015174663.1 PREDICTED: insulin-degrading enzyme isoform X2 [Polistes dominula];XP_015174662.1 PREDICTED: insulin-degrading enzyme isoform X1 [Polistes dominula]	2	3	3	3	118,35	0	15507000	0	0
XP_015174677.1;XP_015174678.1;XP_015174676.1	XP_015174677.1;XP_015174678.1	XP_015174677.1 PREDICTED: ABC transporter F family member 4 isoform X2 [Polistes dominula];XP_015174678.1 PREDICTED: ABC transporter F family member 4 isoform X2 [Polistes dominula]	3	3	3	3	13,019	0	0	0	0
XP_015174730.1	XP_015174730.1	XP_015174730.1 PREDICTED: zinc carboxypeptidase-like [Polistes dominula]	1	3	3	3	46,497	0	95555000	9281100	7502000
XP_015174854.1	XP_015174854.1	XP_015174854.1 PREDICTED: protein disulfide-isomerase A6 [Polistes dominula]	1	3	3	3	47,794	6867400	0	7709100	0
XP_015175030.1	XP_015175030.1	XP_015175030.1 PREDICTED: acylphosphatase-1-like [Polistes dominula]	1	3	3	3	12,4	5014300	51512000	29377000	5687500
XP_015175067.1	XP_015175067.1	XP_015175067.1 PREDICTED: riboflavin kinase, partial [Polistes dominula]	1	3	3	3	16,261	0	57189000	0	0
XP_015175317.1;XP_015175316.1;XP_015175315.1;XP_015175314.1	XP_015175317.1;XP_015175316.1;XP_015175315.1;XP_015175314.1	XP_015175317.1 PREDICTED: matrix metalloproteinase-14 isoform X4 [Polistes dominula];XP_015175316.1 PREDICTED: matrix metalloproteinase-14 isoform X3 [Polistes dominula];XP_015175315.1 PREDICTED: matrix metalloproteinase-14 isoform X2 [Polistes dominula];X	4	3	3	3	59,967	10684000	0	0	0
XP_015176217.1	XP_015176217.1	XP_015176217.1 PREDICTED: succinyl-CoA ligase [ADP/GDP-forming] subunit alpha, mitochondrial [Polistes dominula]	1	3	3	3	35,232	14721000	0	51945000	0
XP_015176676.1;XP_015176675.1	XP_015176676.1;XP_015176675.1	XP_015176676.1 PREDICTED: V-type proton ATPase catalytic subunit A [Polistes dominula];XP_015176675.1 PREDICTED: V-type proton ATPase catalytic subunit A [Polistes dominula]	2	3	3	3	68,342	9161700	0	0	0
XP_015176928.1	XP_015176928.1	XP_015176928.1 PREDICTED: V-type proton ATPase subunit E [Polistes dominula]	1	3	3	3	26,204	22092000	0	0	3290200
XP_015177455.1;XP_015177454.1;XP_015177453.1;XP_015177452.1	XP_015177455.1;XP_015177454.1;XP_015177453.1;XP_015177452.1	XP_015177455.1 PREDICTED: poly(rC)-binding protein 3-like isoform X3 [Polistes dominula];XP_015177454.1 PREDICTED: poly(rC)-binding protein 4-like isoform X2 [Polistes dominula];XP_015177453.1 PREDICTED: poly(rC)-binding protein 4-like isoform X1 [Polistes	4	3	3	3	48,525	0	4666300	0	0
XP_015178431.1;XP_015178430.1;XP_015178429.1;XP_015178428.1;XP_015178427.1	XP_015178431.1;XP_015178430.1;XP_015178429.1;XP_015178428.1;XP_015178427.1	XP_015178431.1 PREDICTED: annexin B11-like isoform X3 [Polistes dominula];XP_015178430.1 PREDICTED: annexin B9-like isoform X2 [Polistes dominula];XP_015178429.1 PREDICTED: annexin B9-like isoform X1 [Polistes dominula];XP_015178428.1 PREDICTED: annexin B9	5	3	3	3	35,709	0	0	0	0
XP_015178474.1	XP_015178474.1	XP_015178474.1 PREDICTED: vanin-like protein 2 [Polistes dominula]	1	3	3	3	59,719	3299100	21862000	0	4314300

XP_015179364.1	XP_015179364.1	XP_015179364.1 PREDICTED: short/branched chain specific acyl-CoA dehydrogenase, mitochondrial [Polistes dominula]	1	3	3	3	45,667	6113600	9774700	0	0
XP_015179424.1	XP_015179424.1	XP_015179424.1 PREDICTED: proliferation-associated protein 2G4 [Polistes dominula]	1	3	3	3	43,499	0	28777000	0	0
XP_015180613.1	XP_015180613.1	XP_015180613.1 PREDICTED: pyridoxine-5-phosphate oxidase-like [Polistes dominula]	1	3	3	3	28,153	0	27627000	4009900	0
XP_015180701.1	XP_015180701.1	XP_015180701.1 PREDICTED: basigin [Polistes dominula]	1	3	3	3	39,836	17263000	12706000	6121500	4015400
XP_015181071.1;XP_015181070.1;XP_015181069.1	XP_015181071.1;XP_015181070.1;XP_015181069.1	XP_015181071.1 PREDICTED: cytosolic non-specific dipeptidase [Polistes dominula];XP_015181070.1 PREDICTED: cytosolic non-specific dipeptidase [Polistes dominula];XP_015181069.1 PREDICTED: cytosolic non-specific dipeptidase [Polistes dominula]	3	3	3	3	54,294	6787400	18981000	11960000	0
XP_015181413.1;XP_015181412.1	XP_015181413.1;XP_015181412.1	XP_015181413.1 PREDICTED: V-type proton ATPase subunit H isoform X2 [Polistes dominula];XP_015181412.1 PREDICTED: V-type proton ATPase subunit H isoform X1 [Polistes dominula]	2	3	3	3	56,338	0	0	0	0
XP_015181488.1	XP_015181488.1	XP_015181488.1 PREDICTED: trypsin-7-like [Polistes dominula]	1	3	3	3	38,836	0	0	0	26620000
XP_015181542.1;XP_015181541.1	XP_015181542.1;XP_015181541.1	XP_015181542.1 PREDICTED: uncharacterized protein LOC107069082 isoform X2 [Polistes dominula];XP_015181541.1 PREDICTED: uncharacterized protein LOC107069082 isoform X1 [Polistes dominula]	2	3	3	3	29,933	0	0	18084000	3922500
XP_015181766.1	XP_015181766.1	XP_015181766.1 PREDICTED: myophilin [Polistes dominula]	1	3	3	3	19,016	0	0	16955000	0
XP_015181900.1	XP_015181900.1	XP_015181900.1 PREDICTED: sepiapterin reductase [Polistes dominula]	1	3	3	3	28,967	18855000	3153700	0	0
XP_015181971.1;XP_015181970.1	XP_015181971.1;XP_015181970.1	XP_015181971.1 PREDICTED: sorbitol dehydrogenase-like isoform X2 [Polistes dominula];XP_015181970.1 PREDICTED: sorbitol dehydrogenase-like isoform X1 [Polistes dominula]	2	3	3	3	36,908	0	0	0	9437500
XP_015182397.1	XP_015182397.1	XP_015182397.1 PREDICTED: putative glucose-6-phosphate 1-epimerase [Polistes dominula]	1	3	3	3	31,847	0	21008000	0	0
XP_015182413.1;XP_015182412.1	XP_015182413.1;XP_015182412.1	XP_015182413.1 PREDICTED: probable cytochrome P450 6a14 [Polistes dominula];XP_015182412.1 PREDICTED: probable cytochrome P450 6a14 [Polistes dominula]	2	3	3	3	59,834	29948000	0	0	0
XP_015182473.1;XP_015182472.1	XP_015182473.1;XP_015182472.1	XP_015182473.1 PREDICTED: actin-5C [Polistes dominula];XP_015182472.1 PREDICTED: actin-5C [Polistes dominula]	2	11	3	3	41,821	144160000	27562000	0	2072600

XP_015182544.1;XP_015182551.1;XP_015182521.1;XP_015182530.1;XP_015182536.1;XP_015182515.1;XP_015182500.1;XP_015182494.1;XP_015182488.1;XP_015182470.1;XP_015182461.1; XP_015182584.1;XP_015182498.1;XP_015182569.1;XP_015182577.1;XP_015182560.1;XP_015182479.1	XP_015182544.1;XP_015182551.1;XP_015182521.1;XP_015182530.1;XP_015182536.1;XP_015182515.1;XP_015182500.1;XP_015182494.1;XP_015182488.1;XP_015182470.1;XP_015182461.1;XP_015182584.1;XP_015182498.1;XP_015182569.1;XP_015182577.1;XP_015182560.1;XP_015182479.1	XP_015182544.1 PREDICTED: tropomyosin isoform X13 [Polistes dominula];XP_015182551.1 PREDICTED: tropomyosin isoform X14 [Polistes dominula];XP_015182521.1 PREDICTED: tropomyosin-2 isoform X10 [Polistes dominula];XP_015182530.1 PREDICTED: tropomyosin-2 isof	17	3	3	3	32,504	2196300	0	0	0
XP_015183214.1	XP_015183214.1	XP_015183214.1 PREDICTED: venom acid phosphatase Acph-1-like [Polistes dominula]	1	3	3	3	46,363	0	0	0	0
XP_015183226.1	XP_015183226.1	XP_015183226.1 PREDICTED: 60S ribosomal protein L23 [Polistes dominula]	1	3	3	3	14,807	6334100	0	16820000	0
XP_015183810.1	XP_015183810.1	XP_015183810.1 PREDICTED: apolipoprotein D-like [Polistes dominula]	1	3	3	3	30,373	29797000	0	0	0
XP_015184178.1	XP_015184178.1	XP_015184178.1 PREDICTED: ras suppressor protein 1 [Polistes dominula]	1	3	3	3	32,089	0	0	0	0
XP_015184240.1	XP_015184240.1	XP_015184240.1 PREDICTED: venom serine protease-like [Polistes dominula]	1	3	3	3	44,044	46897000	0	0	2139400
XP_015184244.1	XP_015184244.1	XP_015184244.1 PREDICTED: trypsin 5G1-like [Polistes dominula]	1	3	3	3	26,338	45855000	0	0	3359800
XP_015184302.1	XP_015184302.1	XP_015184302.1 PREDICTED: calmodulin [Polistes dominula]	1	3	3	3	16,81	13538000	0	0	0
XP_015184723.1	XP_015184723.1	XP_015184723.1 PREDICTED: uncharacterized protein LOC107070756 [Polistes dominula]	1	3	3	3	80,349	13236000	0	0	0

XP_015185451.1;XP_015185371.1;XP_015185302.1;XP_015185217.1;XP_015185139.1;XP_015185047.1;XP_015184970.1;XP_015184896.1	XP_015185451.1;XP_015185371.1;XP_015185302.1;XP_015185217.1;XP_015185139.1;XP_015185047.1;XP_015184970.1;XP_015184896.1	XP_015185451.1 PREDICTED: protein phosphatase 1 regulatory subunit 12A isoform X8 [Polistes dominula];XP_015185371.1 PREDICTED: protein phosphatase 1 regulatory subunit 12A isoform X7 [Polistes dominula];XP_015185302.1 PREDICTED: protein phosphatase 1 regu	8	3	3	3	109,08	0	4018600	0	0
XP_015185363.1;XP_015185362.1	XP_015185363.1;XP_015185362.1	XP_015185363.1 PREDICTED: FK506-binding protein 2 isoform X2 [Polistes dominula];XP_015185362.1 PREDICTED: FK506-binding protein 2 isoform X1 [Polistes dominula]	2	3	3	3	25,897	19797000	0	0	0
XP_015185889.1	XP_015185889.1	XP_015185889.1 PREDICTED: glucose-6-phosphate isomerase [Polistes dominula]	1	3	3	3	62,711	3847500	2137000	0	51414000
XP_015186002.1;XP_015185996.1	XP_015186002.1;XP_015185996.1	XP_015186002.1 PREDICTED: bifunctional glutamate/proline--tRNA ligase isoform X2 [Polistes dominula];XP_015185996.1 PREDICTED: bifunctional glutamate/proline--tRNA ligase isoform X1 [Polistes dominula]	2	3	3	3	175,99	0	82185000	0	0
XP_015186393.1	XP_015186393.1	XP_015186393.1 PREDICTED: LIM and SH3 domain protein Lasp [Polistes dominula]	1	3	3	3	35,947	12352000	3760500	0	0
XP_015186553.1;XP_015186552.1;XP_015186550.1;XP_015186549.1;XP_015186548.1;XP_015186547.1;XP_015186554.1	XP_015186553.1;XP_015186552.1;XP_015186550.1;XP_015186549.1;XP_015186548.1;XP_015186547.1;XP_015186554.1	XP_015186553.1 PREDICTED: 14-3-3 protein zeta isoform X1 [Polistes dominula];XP_015186552.1 PREDICTED: 14-3-3 protein zeta isoform X1 [Polistes dominula];XP_015186550.1 PREDICTED: 14-3-3 protein zeta isoform X1 [Polistes dominula];XP_015186549.1 PREDICTED:	7	3	3	3	28,104	39665000	6688600	0	12162000
XP_015186613.1;XP_015186612.1;XP_015186611.1	XP_015186613.1;XP_015186612.1;XP_015186611.1	XP_015186613.1 PREDICTED: glyoxylate reductase/hydroxypyruvate reductase-like isoform X2 [Polistes dominula];XP_015186612.1 PREDICTED: glyoxylate reductase/hydroxypyruvate reductase-like isoform X2 [Polistes dominula];XP_015186611.1 PREDICTED: glyoxylate r	3	3	3	3	35,557	19656000	12027000	0	5792800
XP_015186656.1;XP_015186657.1;XP_015186659.1	XP_015186656.1;XP_015186657.1;XP_015186659.1	XP_015186656.1 PREDICTED: alpha-crystallin B chain isoform X1 [Polistes dominula];XP_015186657.1 PREDICTED: heat shock protein beta-1 isoform X2 [Polistes dominula];XP_015186659.1 PREDICTED: heat shock protein beta-1 isoform X3 [Polistes dominula]	3	3	3	3	25,593	37506000	0	24707000	5537300
XP_015187286.1;XP_015187285.1;XP_015187283.1;XP_015187282.1;XP_015187281.1;XP_015187280.1	XP_015187286.1;XP_015187285.1;XP_015187283.1;XP_015187282.1;XP_015187281.1;XP_015187280.1	XP_015187286.1 PREDICTED: protein hu-li tai shao isoform X6 [Polistes dominula];XP_015187285.1 PREDICTED: protein hu-li tai shao isoform X5 [Polistes dominula];XP_015187283.1 PREDICTED: protein hu-li tai shao isoform X4 [Polistes dominula];XP_015187282.1 P	6	3	3	3	77,768	3582400	14225000	0	0
XP_015187330.1	XP_015187330.1	XP_015187330.1 PREDICTED: malignant T-cell-amplified sequence 1 homolog [Polistes dominula]	1	3	3	3	20,401	0	23451000	0	0

XP_015187999.1	XP_015187999.1	XP_015187999.1 PREDICTED: trans-1,2-dihydrobenzene-1,2-diol dehydrogenase-like [Polistes dominula]	1	3	3	3	38,199	0	53134000	0	0
XP_015188098.1	XP_015188098.1	XP_015188098.1 PREDICTED: dnaJ protein homolog 1 [Polistes dominula]	1	3	3	3	39,017	0	0	2919800	0
XP_015188943.1	XP_015188943.1	XP_015188943.1 PREDICTED: uncharacterized protein LOC107073050 [Polistes dominula]	1	3	3	3	29,162	5734800	14553000	0	4600000
XP_015189514.1	XP_015189514.1	XP_015189514.1 PREDICTED: neurogenic locus notch homolog protein 1 [Polistes dominula]	1	3	3	3	44,789	0	0	33447000	0
XP_015190415.1;XP_015190414.1;XP_015190413.1;XP_015190412.1	XP_015190415.1;XP_015190414.1;XP_015190413.1;XP_015190412.1	XP_015190415.1 PREDICTED: dnaJ homolog subfamily A member 4 [Polistes dominula];XP_015190414.1 PREDICTED: dnaJ homolog subfamily A member 4 [Polistes dominula];XP_015190413.1 PREDICTED: dnaJ homolog subfamily A member 4 [Polistes dominula];XP_015190412.1 P	4	3	3	3	44,817	3570500	7431100	3952700	3710700
XP_015190627.1;XP_015190626.1	XP_015190627.1;XP_015190626.1	XP_015190627.1 PREDICTED: 3-hydroxyisobutyryl-CoA hydrolase, mitochondrial-like isoform X2 [Polistes dominula];XP_015190626.1 PREDICTED: 3-hydroxyisobutyryl-CoA hydrolase, mitochondrial-like isoform X1 [Polistes dominula]	2	3	3	3	45,178	4999500	39475000	0	4790700
XP_015190745.1;XP_015190744.1	XP_015190745.1;XP_015190744.1	XP_015190745.1 PREDICTED: uncharacterized family 31 glucosidase KIAA1161 isoform X2 [Polistes dominula];XP_015190744.1 PREDICTED: uncharacterized family 31 glucosidase KIAA1161 isoform X1 [Polistes dominula]	2	3	3	3	75,184	0	0	0	43297000
XP_015190972.1	XP_015190972.1	XP_015190972.1 PREDICTED: omega-conotoxin-like protein 1 [Polistes dominula]	1	3	3	3	8,7562	3289100	3322600	0	0
XP_015191187.1;XP_015191186.1;XP_015191185.1	XP_015191187.1;XP_015191186.1;XP_015191185.1	XP_015191187.1 PREDICTED: NECAP-like protein CG9132 [Polistes dominula];XP_015191186.1 PREDICTED: NECAP-like protein CG9132 [Polistes dominula];XP_015191185.1 PREDICTED: NECAP-like protein CG9132 [Polistes dominula]	3	3	3	3	27,079	0	0	0	0
XP_015171465.1;XP_015171464.1	XP_015171465.1;XP_015171464.1	XP_015171465.1 PREDICTED: delta(3,5)-Delta(2,4)-dienoyl-CoA isomerase, mitochondrial [Polistes dominula];XP_015171464.1 PREDICTED: delta(3,5)-Delta(2,4)-dienoyl-CoA isomerase, mitochondrial [Polistes dominula]	2	4	4	4	31,088	9266800	82838000	12478000	6561200
XP_015171842.1;XP_015171841.1	XP_015171842.1;XP_015171841.1	XP_015171842.1 PREDICTED: leucine-rich repeat-containing protein 15-like isoform X2 [Polistes dominula];XP_015171841.1 PREDICTED: leucine-rich repeat-containing protein 15-like isoform X1 [Polistes dominula]	2	4	4	4	75,879	0	6154000	8671600	8084800
XP_015171961.1	XP_015171961.1	XP_015171961.1 PREDICTED: phosphoglycolate phosphatase-like [Polistes dominula]	1	4	4	4	34,436	0	55591000	6671400	0
XP_015172719.1;XP_015172718.1;XP_015172717.1;XP_015172716.1;XP_015172714.1	XP_015172719.1;XP_015172718.1;XP_015172717.1;XP_015172716.1;XP_015172714.1	XP_015172719.1 PREDICTED: oxysterol-binding protein 1 isoform X3 [Polistes dominula];XP_015172718.1 PREDICTED: oxysterol-binding protein 1 isoform X2 [Polistes dominula];XP_015172717.1 PREDICTED: oxysterol-binding protein 1 isoform X1 [Polistes dominula];X	5	4	4	4	84,509	0	0	0	0

XP_015172912.1;XP_015172903.1;XP_015172893.1;XP_015172883.1	XP_015172912.1;XP_015172903.1;XP_015172893.1;XP_015172883.1	XP_015172912.1 PREDICTED: glutathione S-transferase 1-like [Polistes dominula];XP_015172903.1 PREDICTED: glutathione S-transferase 1-like [Polistes dominula];XP_015172893.1 PREDICTED: glutathione S-transferase 1-like [Polistes dominula];XP_015172883.1 PRED	4	4	4	4	24,828	96333000	55809000	0	32735000
XP_015173593.1	XP_015173593.1	XP_015173593.1 PREDICTED: myophilin [Polistes dominula]	1	4	4	4	20,791	7805500	8370600	0	0
XP_015174043.1;XP_015174041.1;XP_015174040.1	XP_015174043.1;XP_015174041.1;XP_015174040.1	XP_015174043.1 PREDICTED: protein yellow-like isoform X2 [Polistes dominula];XP_015174041.1 PREDICTED: protein yellow-like isoform X2 [Polistes dominula];XP_015174040.1 PREDICTED: protein yellow-like isoform X1 [Polistes dominula]	3	4	4	4	49,107	14098000	13711000	0	0
XP_015174620.1;XP_015174619.1;XP_015174618.1;XP_015174617.1	XP_015174620.1;XP_015174619.1;XP_015174618.1;XP_015174617.1	XP_015174620.1 PREDICTED: cAMP-dependent protein kinase type I regulatory subunit isoform X3 [Polistes dominula];XP_015174619.1 PREDICTED: cAMP-dependent protein kinase type I regulatory subunit isoform X2 [Polistes dominula];XP_015174618.1 PREDICTED: cAMP	4	4	4	4	33,304	0	21488000	0	0
XP_015175566.1	XP_015175566.1	XP_015175566.1 PREDICTED: probable trans-2-enoyl-CoA reductase, mitochondrial [Polistes dominula]	1	4	4	4	40,821	13748000	0	0	0
XP_015175695.1	XP_015175695.1	XP_015175695.1 PREDICTED: spermine oxidase-like [Polistes dominula]	1	4	4	4	56,492	0	52831000	9162400	4129800
XP_015175762.1	XP_015175762.1	XP_015175762.1 PREDICTED: cytoplasmic aconitate hydratase-like [Polistes dominula]	1	4	4	4	98,732	0	7772600	0	3053000
XP_015176505.1;XP_015176504.1;XP_015176503.1;XP_015176501.1;XP_015176500.1	XP_015176505.1;XP_015176504.1;XP_015176503.1;XP_015176501.1;XP_015176500.1	XP_015176505.1 PREDICTED: apoptosis-inducing factor 3 isoform X3 [Polistes dominula];XP_015176504.1 PREDICTED: apoptosis-inducing factor 3 isoform X2 [Polistes dominula];XP_015176503.1 PREDICTED: apoptosis-inducing factor 3 isoform X2 [Polistes dominula];X	5	4	4	4	64,251	0	0	0	0
XP_015176576.1;XP_015176575.1;XP_015176574.1	XP_015176576.1;XP_015176575.1;XP_015176574.1	XP_015176576.1 PREDICTED: 40S ribosomal protein S14 [Polistes dominula];XP_015176575.1 PREDICTED: 40S ribosomal protein S14 [Polistes dominula];XP_015176574.1 PREDICTED: 40S ribosomal protein S14 [Polistes dominula]	3	4	4	4	16,205	12273000	45963000	17300000	0

XP_015177045.1;XP_015177047.1;XP_015177028.1;XP_015177029.1;XP_015177032.1;XP_015177033.1;XP_015177034.1;XP_015177035.1;XP_015177036.1;XP_015177037.1;XP_015177038.1; XP_015177039.1;XP_015177040.1;XP_015177041.1;XP_015177043.1;XP_015177044.1;XP_015177046.1;XP_015177027.1;XP_015177030.1	XP_015177045.1;XP_015177047.1;XP_015177028.1;XP_015177029.1;XP_015177032.1;XP_015177033.1;XP_015177034.1;XP_015177035.1;XP_015177036.1;XP_015177037.1;XP_015177038.1;XP_015177039.1;XP_015177040.1;XP_015177041.1;XP_015177043.1;XP_015177044.1;XP_015177046.1; XP_015177027.1;XP_015177030.1	XP_015177045.1 PREDICTED: dystonin isoform X17 [Polistes dominula];XP_015177047.1 PREDICTED: dystonin isoform X19 [Polistes dominula];XP_015177028.1 PREDICTED: microtubule-actin cross-linking factor 1 isoform X2 [Polistes dominula];XP_015177029.1 PREDICTED	19	4	4	4	598,8	0	0	0	0
XP_015177523.1	XP_015177523.1	XP_015177523.1 PREDICTED: late histone H1-like [Polistes dominula]	1	4	4	4	25,418	37400000	99607000	38035000	3108900
XP_015177944.1	XP_015177944.1	XP_015177944.1 PREDICTED: arylphorin subunit beta-like [Polistes dominula]	1	4	4	4	79,11	0	16043000	0	0
XP_015178278.1	XP_015178278.1	XP_015178278.1 PREDICTED: hydroxysteroid dehydrogenase-like protein 2 [Polistes dominula]	1	4	4	4	45,397	12188000	37369000	0	0
XP_015178816.1	XP_015178816.1	XP_015178816.1 PREDICTED: phosphatidylinositol transfer protein alpha isoform [Polistes dominula]	1	4	4	4	31,515	0	0	0	0
XP_015178908.1	XP_015178908.1	XP_015178908.1 PREDICTED: staphylococcal nuclease domain-containing protein 1 [Polistes dominula]	1	4	4	4	100,76	0	39100000	4180900	0
XP_015179032.1;XP_015179034.1;XP_015179033.1	XP_015179032.1;XP_015179034.1;XP_015179033.1	XP_015179032.1 PREDICTED: pleckstrin homology domain-containing family F member 2 isoform X1 [Polistes dominula];XP_015179034.1 PREDICTED: pleckstrin homology domain-containing family F member 2 isoform X3 [Polistes dominula];XP_015179033.1 PREDICTED: plec	3	4	4	4	29,576	0	7913200	0	0
XP_015179360.1;XP_015179362.1;XP_015179361.1;XP_015184119.1	XP_015179360.1	XP_015179360.1 PREDICTED: tubulin alpha-1 chain [Polistes dominula]	4	4	4	4	49,964	16411000	0	0	0
XP_015179363.1	XP_015179363.1	XP_015179363.1 PREDICTED: adenosylhomocysteinase [Polistes dominula]	1	4	4	4	48,027	0	18899000	0	0

XP_015179450.1;XP_015179449.1	XP_015179450.1;XP_015179449.1	XP_015179450.1 PREDICTED: 4-hydroxybutyrate coenzyme A transferase-like [Polistes dominula];XP_015179449.1 PREDICTED: 4-hydroxybutyrate coenzyme A transferase-like [Polistes dominula]	2	4	4	4	52,698	0	245590000	0	0
XP_015179474.1	XP_015179474.1	XP_015179474.1 PREDICTED: TPPP family protein CG45057-like [Polistes dominula]	1	4	4	4	23,334	0	206170000	0	0
XP_015179991.1	XP_015179991.1	XP_015179991.1 PREDICTED: 26S proteasome non-ATPase regulatory subunit 10-like [Polistes dominula]	1	4	4	4	25,026	0	54689000	5225400	0
XP_015181077.1;XP_015181078.1;XP_015188702.1	XP_015181077.1;XP_015181078.1	XP_015181077.1 PREDICTED: ADP-ribosylation factor 1 [Polistes dominula];XP_015181078.1 PREDICTED: ADP-ribosylation factor 1 [Polistes dominula]	3	4	4	4	20,707	15761000	31930000	7516800	0
XP_015181097.1	XP_015181097.1	XP_015181097.1 PREDICTED: pyridoxine/pyridoxamine 5-phosphate oxidase [Polistes dominula]	1	4	4	4	26,068	0	23605000	0	0
XP_015183158.1;XP_015183157.1;XP_015183156.1	XP_015183158.1;XP_015183157.1;XP_015183156.1	XP_015183158.1 PREDICTED: alanine aminotransferase 1 [Polistes dominula];XP_015183157.1 PREDICTED: alanine aminotransferase 1 [Polistes dominula];XP_015183156.1 PREDICTED: alanine aminotransferase 1 [Polistes dominula]	3	4	4	4	60,515	10883000	0	10170000	19787000
XP_015183650.1;XP_015183649.1;XP_015183648.1;XP_015183647.1;XP_015183646.1;XP_015183645.1	XP_015183650.1;XP_015183649.1;XP_015183648.1;XP_015183647.1;XP_015183646.1;XP_015183645.1	XP_015183650.1 PREDICTED: cystathionine gamma-lyase [Polistes dominula];XP_015183649.1 PREDICTED: cystathionine gamma-lyase [Polistes dominula];XP_015183648.1 PREDICTED: cystathionine gamma-lyase [Polistes dominula];XP_015183647.1 PREDICTED: cystathionine	6	4	4	4	42,593	0	39854000	0	2668400
XP_015183815.1;XP_015183814.1;XP_015183813.1;XP_015183811.1	XP_015183815.1;XP_015183814.1;XP_015183813.1;XP_015183811.1	XP_015183815.1 PREDICTED: acidic phospholipase A2 PA4-like isoform X2 [Polistes dominula];XP_015183814.1 PREDICTED: acidic phospholipase A2 PA4-like isoform X1 [Polistes dominula];XP_015183813.1 PREDICTED: acidic phospholipase A2 PA4-like isoform X1 [Polis	4	4	4	4	25,276	1332900000	0	15118000	22399000
XP_015183983.1;XP_015183981.1	XP_015183983.1;XP_015183981.1	XP_015183983.1 PREDICTED: probable cytochrome P450 6a13 [Polistes dominula];XP_015183981.1 PREDICTED: cytochrome P450 6a2-like [Polistes dominula]	2	4	4	4	58,273	21389000	0	0	0
XP_015183992.1	XP_015183992.1	XP_015183992.1 PREDICTED: DNA ligase 1-like [Polistes dominula]	1	4	4	4	76,094	0	21695000	3688200	12484000
XP_015184907.1	XP_015184907.1	XP_015184907.1 PREDICTED: ATPase inhibitor mai-2, mitochondrial-like [Polistes dominula]	1	4	4	4	11,707	18368000	188330000	0	20164000
XP_015186144.1;XP_015186143.1	XP_015186144.1;XP_015186143.1	XP_015186144.1 PREDICTED: carnitine O-acetyltransferase-like isoform X2 [Polistes dominula];XP_015186143.1 PREDICTED: carnitine O-acetyltransferase-like isoform X1 [Polistes dominula]	2	4	4	4	70,658	0	5549400	0	0
XP_015186275.1;XP_015186266.1	XP_015186275.1;XP_015186266.1	XP_015186275.1 PREDICTED: alpha-endosulfine [Polistes dominula];XP_015186266.1 PREDICTED: alpha-endosulfine [Polistes dominula]	2	4	4	4	11,791	0	32328000	0	0
XP_015186377.1;XP_015186378.1	XP_015186377.1;XP_015186378.1	XP_015186377.1 PREDICTED: putative uncharacterized protein DDB_G0282129 isoform X1 [Polistes dominula];XP_015186378.1 PREDICTED: putative uncharacterized protein DDB_G0282129 isoform X2 [Polistes dominula]	2	4	4	4	51,923	4789100	0	0	0

XP_015186479.1;XP_015186478.1	XP_015186479.1;XP_015186478.1	XP_015186479.1 PREDICTED: xaa-Pro dipeptidase isoform X2 [Polistes dominula];XP_015186478.1 PREDICTED: xaa-Pro dipeptidase isoform X1 [Polistes dominula]	2	4	4	4	54,906	0	0	10400000	0
XP_015187218.1;XP_015187217.1;XP_015187215.1;XP_015187214.1	XP_015187218.1;XP_015187217.1;XP_015187215.1;XP_015187214.1	XP_015187218.1 PREDICTED: kynurenine--oxoglutarate transaminase 3 isoform X3 [Polistes dominula];XP_015187217.1 PREDICTED: kynurenine--oxoglutarate transaminase 3 isoform X3 [Polistes dominula];XP_015187215.1 PREDICTED: kynurenine--oxoglutarate transaminas	4	4	4	4	51,131	0	49358000	0	0
XP_015188114.1;XP_015188113.1	XP_015188114.1;XP_015188113.1	XP_015188114.1 PREDICTED: uncharacterized protein LOC107072577 isoform X2 [Polistes dominula];XP_015188113.1 PREDICTED: uncharacterized protein LOC107072577 isoform X1 [Polistes dominula]	2	4	4	4	36,537	165710000	0	31124000	25773000
XP_015188608.1;XP_015188607.1;XP_015188605.1	XP_015188608.1;XP_015188607.1;XP_015188605.1	XP_015188608.1 PREDICTED: pyrroline-5-carboxylate reductase [Polistes dominula];XP_015188607.1 PREDICTED: pyrroline-5-carboxylate reductase [Polistes dominula];XP_015188605.1 PREDICTED: pyrroline-5-carboxylate reductase [Polistes dominula]	3	4	4	4	34,524	54549000	0	0	0
XP_015188854.1	XP_015188854.1	XP_015188854.1 PREDICTED: peroxiredoxin-like [Polistes dominula]	1	5	4	4	26,588	7711500	0	41091000	25534000
XP_015189997.1;XP_015189996.1	XP_015189997.1;XP_015189996.1	XP_015189997.1 PREDICTED: farnesyl pyrophosphate synthase isoform X2 [Polistes dominula];XP_015189996.1 PREDICTED: farnesyl pyrophosphate synthase isoform X1 [Polistes dominula]	2	4	4	4	46,528	4646400	9975500	5265500	2721200
XP_015190507.1;XP_015190499.1	XP_015190507.1;XP_015190499.1	XP_015190507.1 PREDICTED: uncharacterized protein DDB_G0285917-like [Polistes dominula];XP_015190499.1 PREDICTED: uncharacterized protein DDB_G0285917-like [Polistes dominula]	2	4	4	4	26,92	0	0	0	0
XP_015190506.1	XP_015190506.1	XP_015190506.1 PREDICTED: protein lethal(2)essential for life-like [Polistes dominula]	1	4	4	4	20,408	60823000	0	0	0
XP_015190676.1	XP_015190676.1	XP_015190676.1 PREDICTED: beta-hexosaminidase subunit beta-like [Polistes dominula]	1	4	4	4	62,941	0	7616000	0	0
XP_015190851.1;XP_015190850.1;XP_015190849.1	XP_015190851.1;XP_015190850.1;XP_015190849.1	XP_015190851.1 PREDICTED: phospholipase D3-like isoform X2 [Polistes dominula];XP_015190850.1 PREDICTED: phospholipase D3-like isoform X1 [Polistes dominula];XP_015190849.1 PREDICTED: phospholipase D3-like isoform X1 [Polistes dominula]	3	4	4	4	68,374	0	4103000	0	0
XP_015190875.1	XP_015190875.1	XP_015190875.1 PREDICTED: lysosomal Pro-X carboxypeptidase [Polistes dominula]	1	4	4	4	57,11	49236000	0	0	0
XP_015171438.1;XP_015171430.1;XP_015171422.1	XP_015171438.1;XP_015171430.1;XP_015171422.1	XP_015171438.1 PREDICTED: protein-glutamate O-methyltransferase-like isoform X3 [Polistes dominula];XP_015171430.1 PREDICTED: protein-glutamate O-methyltransferase-like isoform X2 [Polistes dominula];XP_015171422.1 PREDICTED: protein-glutamate O-methyltran	3	5	5	5	50,726	0	41937000	0	0
XP_015171536.1	XP_015171536.1	XP_015171536.1 PREDICTED: cAMP-dependent protein kinase catalytic subunit [Polistes dominula]	1	5	5	5	40,783	0	13369000	0	0
XP_015171552.1	XP_015171552.1	XP_015171552.1 PREDICTED: ubiquitin-conjugating enzyme E2 L3 [Polistes dominula]	1	5	5	5	18,131	11291000	105090000	6216300	20221000
XP_015171740.1	XP_015171740.1	XP_015171740.1 PREDICTED: probable methylmalonate-semialdehyde dehydrogenase [acylating], mitochondrial [Polistes dominula]	1	5	5	5	56,389	41571000	0	0	0
XP_015172171.1	XP_015172171.1	XP_015172171.1 PREDICTED: phosphoglycerate kinase [Polistes dominula]	1	5	5	5	44,737	8717300	0	0	19521000
XP_015172505.1	XP_015172505.1	XP_015172505.1 PREDICTED: ester hydrolase C11orf54 homolog [Polistes dominula]	1	5	5	5	35,472	8483600	150070000	48839000	14845000

XP_015172622.1	XP_015172622.1	XP_015172622.1 PREDICTED: 3-hydroxyacyl-CoA dehydrogenase type-2-like [Polistes dominula]	1	7	5	5	27,095	43105000	130710000	44170000	43229000
XP_015173767.1;XP_015173766.1;XP_015173765.1	XP_015173767.1;XP_015173766.1;XP_015173765.1	XP_015173767.1 PREDICTED: membrane metallo-endopeptidase-like 1 isoform X3 [Polistes dominula];XP_015173766.1 PREDICTED: neprilysin isoform X2 [Polistes dominula];XP_015173765.1 PREDICTED: neprilysin isoform X1 [Polistes dominula]	3	5	5	5	86,49	45275000	0	20402000	0
XP_015173946.1;XP_015173945.1;XP_015173944.1;XP_015173943.1	XP_015173946.1;XP_015173945.1;XP_015173944.1;XP_015173943.1	XP_015173946.1 PREDICTED: calcium-transporting ATPase sarcoplasmic/endoplasmic reticulum type isoform X2 [Polistes dominula];XP_015173945.1 PREDICTED: calcium-transporting ATPase sarcoplasmic/endoplasmic reticulum type isoform X1 [Polistes dominula];XP_015	4	5	5	5	109,93	2756200	0	0	151220000
XP_015174104.1	XP_015174104.1	XP_015174104.1 PREDICTED: eukaryotic translation initiation factor 2 subunit 3, Y-linked [Polistes dominula]	1	5	5	5	51,347	0	0	0	0
XP_015175452.1	XP_015175452.1	XP_015175452.1 PREDICTED: ubiquitin-like modifier-activating enzyme 1 [Polistes dominula]	1	5	5	5	117,32	0	0	0	0
XP_015175839.1;XP_015175838.1	XP_015175839.1;XP_015175838.1	XP_015175839.1 PREDICTED: BAG domain-containing protein Samui isoform X2 [Polistes dominula];XP_015175838.1 PREDICTED: BAG domain-containing protein Samui isoform X1 [Polistes dominula]	2	5	5	5	79,098	0	98502000	11092000	4940200
XP_015176246.1	XP_015176246.1	XP_015176246.1 PREDICTED: transmembrane protease serine 9-like [Polistes dominula]	1	5	5	5	88,322	0	111410000	27037000	123690000
XP_015176334.1	XP_015176334.1	XP_015176334.1 PREDICTED: LOW QUALITY PROTEIN: protein Red-like [Polistes dominula]	1	5	5	5	100,96	1606400	56913000	0	6110000
XP_015176808.1	XP_015176808.1	XP_015176808.1 PREDICTED: bleomycin hydrolase [Polistes dominula]	1	5	5	5	52,087	0	0	0	10929000
XP_015176815.1	XP_015176815.1	XP_015176815.1 PREDICTED: lambda-crystallin homolog [Polistes dominula]	1	5	5	5	35,361	2743000	0	0	19621000
XP_015176830.1;XP_015176829.1	XP_015176830.1;XP_015176829.1	XP_015176830.1 PREDICTED: fumarate hydratase, mitochondrial-like isoform X2 [Polistes dominula];XP_015176829.1 PREDICTED: fumarate hydratase, mitochondrial-like isoform X1 [Polistes dominula]	2	5	5	5	51,303	4760200	0	0	50277000
XP_015176921.1;XP_015176920.1;XP_015176919.1	XP_015176921.1;XP_015176920.1;XP_015176919.1	XP_015176921.1 PREDICTED: fructose-1,6-bisphosphatase 1 [Polistes dominula];XP_015176920.1 PREDICTED: fructose-1,6-bisphosphatase 1 [Polistes dominula];XP_015176919.1 PREDICTED: fructose-1,6-bisphosphatase 1 [Polistes dominula]	3	5	5	5	37,122	0	11730000	7093700	0
XP_015177498.1;XP_015177497.1;XP_015177496.1;XP_015177495.1	XP_015177498.1;XP_015177497.1;XP_015177496.1;XP_015177495.1	XP_015177498.1 PREDICTED: xaa-Pro aminopeptidase 1-like isoform X2 [Polistes dominula];XP_015177497.1 PREDICTED: xaa-Pro aminopeptidase 1-like isoform X2 [Polistes dominula];XP_015177496.1 PREDICTED: xaa-Pro aminopeptidase 1-like isoform X2 [Polistes domin	4	5	5	5	70,221	0	55300000	0	0
XP_015179219.1	XP_015179219.1	XP_015179219.1 PREDICTED: phosphoacetylglucosamine mutase [Polistes dominula]	1	5	5	5	60,477	0	3122700	0	0
XP_015180221.1;XP_015180220.1;XP_015180218.1;XP_015180222.1	XP_015180221.1;XP_015180220.1;XP_015180218.1;XP_015180222.1	XP_015180221.1 PREDICTED: probable serine/threonine-protein kinase clkA isoform X1 [Polistes dominula];XP_015180220.1 PREDICTED: probable serine/threonine-protein kinase clkA isoform X1 [Polistes dominula];XP_015180218.1 PREDICTED: probable serine/threonin	4	5	5	5	32,4	26176000	30528000	3463200	4697800
XP_015180240.1	XP_015180240.1	XP_015180240.1 PREDICTED: succinyl-CoA:3-ketoacid coenzyme A transferase 1, mitochondrial [Polistes dominula]	1	5	5	5	58,542	0	23267000	0	0

XP_015180297.1;XP_015180296.1	XP_015180297.1;XP_015180296.1	XP_015180297.1 PREDICTED: UPF0553 protein C9orf64 homolog isoform X2 [Polistes dominula];XP_015180296.1 PREDICTED: UPF0553 protein C9orf64 homolog isoform X1 [Polistes dominula]	2	5	5	5	36,939	0	16346000	0	4855100
XP_015180729.1	XP_015180729.1	XP_015180729.1 PREDICTED: ejaculatory bulb-specific protein 3 [Polistes dominula]	1	5	5	5	14,158	26915000	153680000	22652000	0
XP_015180779.1	XP_015180779.1	XP_015180779.1 PREDICTED: enolase [Polistes dominula]	1	5	5	5	46,982	29093000	67668000	0	190000000
XP_015181250.1;XP_015181249.1;XP_015181248.1	XP_015181250.1;XP_015181249.1;XP_015181248.1	XP_015181250.1 PREDICTED: protein D2-like isoform X2 [Polistes dominula];XP_015181249.1 PREDICTED: protein D2-like isoform X2 [Polistes dominula];XP_015181248.1 PREDICTED: protein D2-like isoform X1 [Polistes dominula]	3	5	5	5	20,796	23208000	18492000	0	0
XP_015181294.1	XP_015181294.1	XP_015181294.1 PREDICTED: farnesol dehydrogenase-like [Polistes dominula]	1	5	5	4	27,878	259130000	0	0	2230100
XP_015182808.1	XP_015182808.1	XP_015182808.1 PREDICTED: protein SGT1 homolog [Polistes dominula]	1	5	5	5	24,431	0	26793000	0	0
XP_015182865.1	XP_015182865.1	XP_015182865.1 PREDICTED: thioredoxin-2 [Polistes dominula]	1	5	5	5	12,084	169240000	146170000	196000000	174530000
XP_015182997.1;XP_015182996.1	XP_015182997.1;XP_015182996.1	XP_015182997.1 PREDICTED: lactoylglutathione lyase [Polistes dominula];XP_015182996.1 PREDICTED: lactoylglutathione lyase [Polistes dominula]	2	5	5	5	20,973	0	60860000	0	10503000
XP_015183291.1;XP_015183290.1;XP_015183295.1;XP_015183294.1;XP_015183292.1;XP_015183296.1;XP_015183293.1	XP_015183291.1;XP_015183290.1;XP_015183295.1;XP_015183294.1;XP_015183292.1;XP_015183296.1;XP_015183293.1	XP_015183291.1 PREDICTED: twitchin isoform X2 [Polistes dominula];XP_015183290.1 PREDICTED: twitchin isoform X1 [Polistes dominula];XP_015183295.1 PREDICTED: twitchin isoform X6 [Polistes dominula];XP_015183294.1 PREDICTED: twitchin isoform X5 [Polistes do	7	25	5	5	872,58	0	28842000	19581000	0
XP_015183315.1	XP_015183315.1	XP_015183315.1 PREDICTED: uncharacterized protein LOC107070028 [Polistes dominula]	1	5	5	3	118,77	0	0	0	37101000
XP_015183694.1;XP_015183693.1;XP_015183692.1;XP_015183691.1;XP_015183689.1;XP_015183688.1;XP_015183687.1	XP_015183694.1;XP_015183693.1;XP_015183692.1;XP_015183691.1;XP_015183689.1;XP_015183688.1;XP_015183687.1	XP_015183694.1 PREDICTED: spectrin beta chain isoform X5 [Polistes dominula];XP_015183693.1 PREDICTED: spectrin beta chain isoform X4 [Polistes dominula];XP_015183692.1 PREDICTED: spectrin beta chain isoform X3 [Polistes dominula];XP_015183691.1 PREDICTED:	7	5	5	5	266,11	29203000	5943000	0	6698000
XP_015184245.1	XP_015184245.1	XP_015184245.1 PREDICTED: acyl-CoA synthetase family member 2, mitochondrial [Polistes dominula]	1	5	5	5	67,886	63711000	0	0	0

XP_015186858.1;XP_015186856.1;XP_015186855.1;XP_015186854.1;XP_015186853.1	XP_015186858.1;XP_015186856.1;XP_015186855.1;XP_015186854.1;XP_015186853.1	XP_015186858.1 PREDICTED: annexin B9-like isoform X1 [Polistes dominula];XP_015186856.1 PREDICTED: annexin B9-like isoform X1 [Polistes dominula];XP_015186855.1 PREDICTED: annexin B9-like isoform X3 [Polistes dominula];XP_015186854.1 PREDICTED: annexin B9-	5	5	5	5	35,687	46905000	0	0	0
XP_015187249.1	XP_015187249.1	XP_015187249.1 PREDICTED: venom serine carboxypeptidase [Polistes dominula]	1	5	5	5	53,443	23592000	10662000	23722000	0
XP_015187603.1	XP_015187603.1	XP_015187603.1 PREDICTED: uncharacterized protein LOC107072304 [Polistes dominula]	1	5	5	5	103,15	0	55843000	0	0
XP_015187733.1	XP_015187733.1	XP_015187733.1 PREDICTED: carbonyl reductase [NADPH] 1-like [Polistes dominula]	1	5	5	5	30,552	22627000	0	0	43968000
XP_015187959.1	XP_015187959.1	XP_015187959.1 PREDICTED: dihydrolipoyllysine-residue succinyltransferase component of 2-oxoglutarate dehydrogenase complex, mitochondrial [Polistes dominula]	1	5	5	5	52,973	0	0	45234000	4212000
XP_015171793.1	XP_015171793.1	XP_015171793.1 PREDICTED: dehydrogenase/reductase SDR family member 11-like [Polistes dominula]	1	6	6	6	28,411	110010000	2285100	0	31056000
XP_015173092.1	XP_015173092.1	XP_015173092.1 PREDICTED: protein lethal(2)essential for life-like [Polistes dominula]	1	6	6	6	23,025	174320000	63369000	61669000	17503000
XP_015173221.1	XP_015173221.1	XP_015173221.1 PREDICTED: maltase 1-like [Polistes dominula]	1	6	6	5	65,465	0	13518000	0	134720000
XP_015173434.1;XP_015173433.1;XP_015173431.1;XP_015173430.1	XP_015173434.1;XP_015173433.1;XP_015173431.1;XP_015173430.1	XP_015173434.1 PREDICTED: amphiphysin isoform X4 [Polistes dominula];XP_015173433.1 PREDICTED: myc box-dependent-interacting protein 1 isoform X3 [Polistes dominula];XP_015173431.1 PREDICTED: amphiphysin isoform X2 [Polistes dominula];XP_015173430.1 PREDIC	4	6	6	6	40,284	0	144810000	0	5503900
XP_015174380.1	XP_015174380.1	XP_015174380.1 PREDICTED: multifunctional protein ADE2 [Polistes dominula]	1	6	6	6	47,445	0	8677900	0	0
XP_015174956.1	XP_015174956.1	XP_015174956.1 PREDICTED: 10 kDa heat shock protein, mitochondrial [Polistes dominula]	1	6	6	6	11,56	353500000	2895400	34303000	0
XP_015175459.1	XP_015175459.1	XP_015175459.1 PREDICTED: superoxide dismutase [Cu-Zn] [Polistes dominula]	1	6	6	6	15,87	105300000	8636200	15966000	34609000
XP_015175500.1	XP_015175500.1	XP_015175500.1 PREDICTED: chymotrypsin-2-like [Polistes dominula]	1	6	6	6	27,119	0	42271000	0	25628000
XP_015177428.1;XP_015177419.1;XP_015177411.1;XP_015177404.1;XP_015177397.1	XP_015177428.1;XP_015177419.1;XP_015177411.1;XP_015177404.1;XP_015177397.1	XP_015177428.1 PREDICTED: adenylate kinase isoenzyme 1 [Polistes dominula];XP_015177419.1 PREDICTED: adenylate kinase isoenzyme 1 [Polistes dominula];XP_015177411.1 PREDICTED: adenylate kinase isoenzyme 1 [Polistes dominula];XP_015177404.1 PREDICTED: adeny	5	6	6	6	21,027	0	51760000	81669000	0
XP_015177545.1;XP_015177544.1	XP_015177545.1;XP_015177544.1	XP_015177545.1 PREDICTED: leukotriene A-4 hydrolase [Polistes dominula];XP_015177544.1 PREDICTED: leukotriene A-4 hydrolase [Polistes dominula]	2	6	6	6	70,308	0	86513000	7728100	10078000
XP_015178193.1	XP_015178193.1	XP_015178193.1 PREDICTED: lysosomal alpha-mannosidase [Polistes dominula]	1	6	6	6	194,5	0	6207800	0	0

XP_015178720.1	XP_015178720.1	XP_015178720.1 PREDICTED: 3-hydroxyisobutyryl-CoA hydrolase, mitochondrial-like [Polistes dominula]	1	6	6	6	47,201	51900000	5793600	0	0
XP_015179926.1	XP_015179926.1	XP_015179926.1 PREDICTED: elongation factor 1-gamma [Polistes dominula]	1	6	6	6	49,14	11969000	26022000	20424000	5496000
XP_015181085.1;XP_015181084.1	XP_015181085.1;XP_015181084.1	XP_015181085.1 PREDICTED: hydroxymethylglutaryl-CoA synthase 1 [Polistes dominula];XP_015181084.1 PREDICTED: hydroxymethylglutaryl-CoA synthase 1 [Polistes dominula]	2	6	6	6	51,505	64770000	0	0	0
XP_015181200.1	XP_015181200.1	XP_015181200.1 PREDICTED: adenylate kinase [Polistes dominula]	1	6	6	6	27,929	47333000	36940000	12327000	4938900
XP_015182542.1	XP_015182542.1	XP_015182542.1 PREDICTED: uncharacterized protein LOC107069607 [Polistes dominula]	1	6	6	6	58,853	0	88854000	20698000	0
XP_015183659.1;XP_015183658.1	XP_015183659.1;XP_015183658.1	XP_015183659.1 PREDICTED: 6-phosphogluconate dehydrogenase, decarboxylating isoform X2 [Polistes dominula];XP_015183658.1 PREDICTED: 6-phosphogluconate dehydrogenase, decarboxylating isoform X1 [Polistes dominula]	2	6	6	6	53,389	63744000	0	0	4174200
XP_015184549.1	XP_015184549.1	XP_015184549.1 PREDICTED: peptidyl-prolyl cis-trans isomerase-like [Polistes dominula]	1	6	6	6	23,076	0	0	0	9631100
XP_015184850.1	XP_015184850.1	XP_015184850.1 PREDICTED: selenium-binding protein 1 [Polistes dominula]	1	6	6	6	47,25	97554000	0	0	4596500
XP_015186960.1;XP_015189987.1;XP_015189952.1	XP_015186960.1	XP_015186960.1 PREDICTED: endochitinase EP3-like [Polistes dominula]	3	6	6	6	21,112	0	486750000	122140000	44652000
XP_015187147.1;XP_015187146.1	XP_015187147.1;XP_015187146.1	XP_015187147.1 PREDICTED: endoplasmic reticulum resident protein 29 [Polistes dominula];XP_015187146.1 PREDICTED: endoplasmic reticulum resident protein 29 [Polistes dominula]	2	6	6	6	28,055	0	3332400	6183700	0
XP_015187740.1	XP_015187740.1	XP_015187740.1 PREDICTED: juvenile hormone epoxide hydrolase 1-like, partial [Polistes dominula]	1	6	6	6	54,223	85658000	0	0	0
XP_015188866.1;XP_015188867.1	XP_015188866.1;XP_015188867.1	XP_015188866.1 PREDICTED: rho GDP-dissociation inhibitor 2 isoform X1 [Polistes dominula];XP_015188867.1 PREDICTED: dynein assembly factor 5, axonemal isoform X2 [Polistes dominula]	2	6	6	6	24,298	0	317740000	0	0
XP_015190183.1	XP_015190183.1	XP_015190183.1 PREDICTED: peptidyl-prolyl cis-trans isomerase 5 [Polistes dominula]	1	6	6	6	27,415	149280000	613620000	0	54851000

XP_015190290.1;XP_015190292.1;XP_015190281.1;XP_015190274.1;XP_015190275.1;XP_015190276.1;XP_015190277.1;XP_015190278.1;XP_015190279.1;XP_015190280.1;XP_015190282.1;XP_015190291.1;XP_015190283.1;XP_015190284.1;XP_015190286.1;XP_015190287.1;XP_015190288.1;XP_015190289.1;XP_015190273.1	XP_015190290.1;XP_015190292.1;XP_015190281.1;XP_015190274.1;XP_015190275.1;XP_015190276.1;XP_015190277.1;XP_015190278.1;XP_015190279.1;XP_015190280.1;XP_015190282.1;XP_015190291.1;XP_015190283.1;XP_015190284.1;XP_015190286.1;XP_015190287.1;XP_015190288.1;XP_015190289.1;XP_015190273.1	XP_015190290.1 PREDICTED: serine protease inhibitor 3/4-like isoform X17 [Polistes dominula];XP_015190292.1 PREDICTED: serine protease inhibitor 3/4-like isoform X19 [Polistes dominula];XP_015190281.1 PREDICTED: serine protease inhibitor 3/4-like isoform X	19	6	6	6	43,924	42241000	77614000	14591000	24512000
XP_015191429.1	XP_015191429.1	XP_015191429.1 PREDICTED: glucosylceramidase [Polistes dominula]	1	6	6	6	61,21	50921000	0	0	3414900
XP_015191446.1	XP_015191446.1	XP_015191446.1 PREDICTED: alpha-aminoadipic semialdehyde dehydrogenase [Polistes dominula]	1	6	6	6	57,199	0	4929600	4558000	0
XP_015171188.1;XP_015171189.1	XP_015171188.1;XP_015171189.1	XP_015171188.1 PREDICTED: pterin-4-alpha-carbinolamine dehydratase isoform X1 [Polistes dominula];XP_015171189.1 PREDICTED: pterin-4-alpha-carbinolamine dehydratase isoform X2 [Polistes dominula]	2	7	7	7	16,438	30289000	374050000	10037000	0
XP_015173496.1;XP_015173495.1	XP_015173496.1;XP_015173495.1	XP_015173496.1 PREDICTED: ribonuclease Oy [Polistes dominula];XP_015173495.1 PREDICTED: ribonuclease Oy [Polistes dominula]	2	7	7	7	29,623	97594000	3420600	2371100	18990000
XP_015175719.1	XP_015175719.1	XP_015175719.1 PREDICTED: phosphoglycerate mutase 2 [Polistes dominula]	1	7	7	7	28,712	0	159410000	0	30493000
XP_015176784.1	XP_015176784.1	XP_015176784.1 PREDICTED: venom carboxylesterase-6-like [Polistes dominula]	1	7	7	7	63,811	0	16191000	98690000	0
XP_015177560.1	XP_015177560.1	XP_015177560.1 PREDICTED: ATP synthase subunit alpha, mitochondrial [Polistes dominula]	1	7	7	7	59,518	127100000	0	0	0
XP_015178440.1	XP_015178440.1	XP_015178440.1 PREDICTED: group XV phospholipase A2-like [Polistes dominula]	1	7	7	7	46,645	42533000	2908000	0	2242900
XP_015178665.1;XP_015178664.1	XP_015178665.1;XP_015178664.1	XP_015178665.1 PREDICTED: 97 kDa heat shock protein isoform X2 [Polistes dominula];XP_015178664.1 PREDICTED: 97 kDa heat shock protein isoform X1 [Polistes dominula]	2	7	7	7	93,427	22816000	13319000	4898600	0

XP_015178840.1;XP_015178839.1;XP_015178838.1;XP_015178835.1;XP_015178842.1;XP_015178837.1;XP_015178836.1;XP_015178843.1	XP_015178840.1;XP_015178839.1;XP_015178838.1;XP_015178835.1;XP_015178842.1;XP_015178837.1;XP_015178836.1;XP_015178843.1	XP_015178840.1 PREDICTED: PDZ and LIM domain protein 3 isoform X6 [Polistes dominula];XP_015178839.1 PREDICTED: PDZ and LIM domain protein 3 isoform X5 [Polistes dominula];XP_015178838.1 PREDICTED: PDZ and LIM domain protein 3 isoform X4 [Polistes dominula	8	7	7	7	36,262	0	114230000	183620000	16177000
XP_015178862.1;XP_015178864.1;XP_015178863.1	XP_015178862.1;XP_015178864.1;XP_015178863.1	XP_015178862.1 PREDICTED: transketolase-like protein 2 isoform X1 [Polistes dominula];XP_015178864.1 PREDICTED: transketolase-like protein 2 isoform X2 [Polistes dominula];XP_015178863.1 PREDICTED: transketolase-like protein 2 isoform X2 [Polistes dominula	3	7	7	7	67,984	76747000	0	0	0
XP_015178907.1	XP_015178907.1	XP_015178907.1 PREDICTED: lysosomal aspartic protease [Polistes dominula]	1	7	7	7	42,041	35717000	0	48907000	0
XP_015179615.1	XP_015179615.1	XP_015179615.1 PREDICTED: uncharacterized oxidoreductase YjmC [Polistes dominula]	1	7	7	7	44,634	102600000	0	0	0
XP_015179941.1	XP_015179941.1	XP_015179941.1 PREDICTED: 4-coumarate--CoA ligase 1 [Polistes dominula]	1	7	7	7	66,64	103730000	0	0	0
XP_015180271.1;XP_015180270.1;XP_015180269.1;XP_015180268.1;XP_015180267.1;XP_015180266.1;XP_015180265.1;XP_015180264.1	XP_015180271.1;XP_015180270.1;XP_015180269.1;XP_015180268.1;XP_015180267.1;XP_015180266.1;XP_015180265.1;XP_015180264.1	XP_015180271.1 PREDICTED: muscle M-line assembly protein unc-89 isoform X8 [Polistes dominula];XP_015180270.1 PREDICTED: muscle M-line assembly protein unc-89 isoform X7 [Polistes dominula];XP_015180269.1 PREDICTED: muscle M-line assembly protein unc-89 is	8	7	7	7	606,68	0	6682400	0	15741000
XP_015180646.1	XP_015180646.1	XP_015180646.1 PREDICTED: glycogen phosphorylase [Polistes dominula]	1	7	7	7	97,461	0	0	0	84646000
XP_015180934.1;XP_015180933.1;XP_015180932.1	XP_015180934.1;XP_015180933.1;XP_015180932.1	XP_015180934.1 PREDICTED: NADP-dependent malic enzyme isoform X2 [Polistes dominula];XP_015180933.1 PREDICTED: NADP-dependent malic enzyme isoform X1 [Polistes dominula];XP_015180932.1 PREDICTED: NADP-dependent malic enzyme isoform X1 [Polistes dominula]	3	7	7	7	68,391	0	0	5764100	60683000
XP_015181315.1	XP_015181315.1	XP_015181315.1 PREDICTED: uncharacterized protein LOC107068952 [Polistes dominula]	1	7	7	7	19,253	839040000	23077000	47906000	84305000
XP_015181406.1	XP_015181406.1	XP_015181406.1 PREDICTED: peptidyl-prolyl cis-trans isomerase [Polistes dominula]	1	7	7	7	18,063	33094000	52859000	8978700	47713000

XP_015181584.1;XP_015181583.1;XP_015181582.1;XP_015181581.1;XP_015181580.1;XP_015181579.1;XP_015181578.1;XP_015181576.1	XP_015181584.1;XP_015181583.1;XP_015181582.1;XP_015181581.1;XP_015181580.1;XP_015181579.1;XP_015181578.1;XP_015181576.1	XP_015181584.1 PREDICTED: alpha-N-acetylgalactosaminidase isoform X2 [Polistes dominula];XP_015181583.1 PREDICTED: alpha-N-acetylgalactosaminidase isoform X2 [Polistes dominula];XP_015181582.1 PREDICTED: alpha-N-acetylgalactosaminidase isoform X2 [Polistes	8	7	7	7	50,344	0	32199000	0	54354000
XP_015182528.1	XP_015182528.1	XP_015182528.1 PREDICTED: nuclear migration protein nudC [Polistes dominula]	1	7	7	7	38,593	0	86404000	0	0
XP_015182852.1;XP_015182851.1;XP_015182850.1	XP_015182852.1;XP_015182851.1;XP_015182850.1	XP_015182852.1 PREDICTED: digestive cysteine proteinase 1 [Polistes dominula];XP_015182851.1 PREDICTED: digestive cysteine proteinase 1 [Polistes dominula];XP_015182850.1 PREDICTED: digestive cysteine proteinase 1 [Polistes dominula]	3	7	7	7	62,048	49251000	34157000	20767000	0
XP_015184110.1	XP_015184110.1	XP_015184110.1 PREDICTED: rab GDP dissociation inhibitor beta [Polistes dominula]	1	7	7	7	50,023	0	87013000	0	0
XP_015184671.1;XP_015184669.1;XP_015184668.1	XP_015184671.1;XP_015184669.1;XP_015184668.1	XP_015184671.1 PREDICTED: trehalase-like isoform X2 [Polistes dominula];XP_015184669.1 PREDICTED: trehalase-like isoform X1 [Polistes dominula];XP_015184668.1 PREDICTED: trehalase-like isoform X1 [Polistes dominula]	3	7	7	7	68,068	25180000	49119000	0	8939100
XP_015187116.1	XP_015187116.1	XP_015187116.1 PREDICTED: phospholipase A1-like [Polistes dominula]	1	8	7	7	37,852	403260000	0	131100000	170700000
XP_015187795.1;XP_015187794.1;XP_015187793.1;XP_015187796.1	XP_015187795.1;XP_015187794.1;XP_015187793.1;XP_015187796.1	XP_015187795.1 PREDICTED: glucose-6-phosphate 1-dehydrogenase isoform X3 [Polistes dominula];XP_015187794.1 PREDICTED: glucose-6-phosphate 1-dehydrogenase isoform X2 [Polistes dominula];XP_015187793.1 PREDICTED: glucose-6-phosphate 1-dehydrogenase isoform	4	7	7	7	60,237	44026000	0	0	0
XP_015189376.1	XP_015189376.1	XP_015189376.1 PREDICTED: eukaryotic translation initiation factor 2A [Polistes dominula]	1	7	7	7	68,431	0	31750000	0	0
XP_015190124.1;XP_015190123.1	XP_015190124.1;XP_015190123.1	XP_015190124.1 PREDICTED: probable phospholipid hydroperoxide glutathione peroxidase isoform X2 [Polistes dominula];XP_015190123.1 PREDICTED: probable phospholipid hydroperoxide glutathione peroxidase isoform X1 [Polistes dominula]	2	7	7	7	21,926	19199000	142560000	99320000	9543200
XP_015191284.1	XP_015191284.1	XP_015191284.1 PREDICTED: GTP:AMP phosphotransferase AK3, mitochondrial [Polistes dominula]	1	7	7	7	24,975	341620000	0	0	0
XP_015191468.1	XP_015191468.1	XP_015191468.1 PREDICTED: peroxisomal multifunctional enzyme type 2-like [Polistes dominula]	1	7	7	7	79,329	32832000	0	0	2223300
XP_015191652.1;XP_015191651.1;XP_015191650.1;XP_015191649.1	XP_015191652.1;XP_015191651.1;XP_015191650.1;XP_015191649.1	XP_015191652.1 PREDICTED: uncharacterized protein LOC107074598 isoform X3 [Polistes dominula];XP_015191651.1 PREDICTED: uncharacterized protein LOC107074598 isoform X3 [Polistes dominula];XP_015191650.1 PREDICTED: uncharacterized protein LOC107074598 isofo	4	7	7	7	46,925	0	72135000	0	5230000
XP_015173889.1	XP_015173889.1	XP_015173889.1 PREDICTED: peroxisomal hydratase-dehydrogenase-epimerase-like [Polistes dominula]	1	8	8	8	94,529	9901500	104830000	70677000	32068000

XP_015176466.1;XP_015176485.1;XP_015176476.1;XP_015176502.1;XP_015176494.1	XP_015176466.1;XP_015176485.1;XP_015176476.1;XP_015176502.1;XP_015176494.1	XP_015176466.1 PREDICTED: muscle LIM protein Mlp84B-like isoform X1 [Polistes dominula];XP_015176485.1 PREDICTED: muscle LIM protein 1-like isoform X3 [Polistes dominula];XP_015176476.1 PREDICTED: muscle LIM protein Mlp84B-like isoform X2 [Polistes dominul	5	8	8	8	53,388	0	694730000	3527600000	37654000
XP_015176638.1;XP_015176637.1;XP_015176636.1;XP_015176634.1	XP_015176638.1;XP_015176637.1;XP_015176636.1;XP_015176634.1	XP_015176638.1 PREDICTED: hexokinase type 2 isoform X4 [Polistes dominula];XP_015176637.1 PREDICTED: hexokinase type 2 isoform X3 [Polistes dominula];XP_015176636.1 PREDICTED: hexokinase type 2 isoform X2 [Polistes dominula];XP_015176634.1 PREDICTED: hexok	4	8	8	8	49,86	0	33111000	0	0
XP_015178632.1;XP_015178631.1;XP_015178630.1;XP_015178628.1;XP_015178629.1	XP_015178632.1;XP_015178631.1;XP_015178630.1;XP_015178628.1;XP_015178629.1	XP_015178632.1 PREDICTED: microtubule-associated protein tau-like isoform X5 [Polistes dominula];XP_015178631.1 PREDICTED: microtubule-associated protein 4-like isoform X4 [Polistes dominula];XP_015178630.1 PREDICTED: microtubule-associated protein 4-like	5	8	8	8	125,78	8435800	16897000	4666000	2057800
XP_015181623.1;XP_015181629.1;XP_015181628.1;XP_015181627.1;XP_015181626.1;XP_015181625.1;XP_015181624.1	XP_015181623.1;XP_015181629.1;XP_015181628.1;XP_015181627.1;XP_015181626.1;XP_015181625.1;XP_015181624.1	XP_015181623.1 PREDICTED: moesin/ezrin/radixin homolog 1 isoform X1 [Polistes dominula];XP_015181629.1 PREDICTED: moesin/ezrin/radixin homolog 1 isoform X7 [Polistes dominula];XP_015181628.1 PREDICTED: moesin/ezrin/radixin homolog 1 isoform X6 [Polistes do	7	8	8	8	60,225	38699000	28268000	0	5872500
XP_015183406.1;XP_015183405.1;XP_015183404.1;XP_015183403.1;XP_015183402.1;XP_015183401.1	XP_015183406.1;XP_015183405.1;XP_015183404.1;XP_015183403.1;XP_015183402.1;XP_015183401.1	XP_015183406.1 PREDICTED: adenylyl cyclase-associated protein 1 isoform X4 [Polistes dominula];XP_015183405.1 PREDICTED: adenylyl cyclase-associated protein 1 isoform X3 [Polistes dominula];XP_015183404.1 PREDICTED: adenylyl cyclase-associated protein 1 is	6	8	8	8	56,943	6878000	546000000	52958000	16682000
XP_015183663.1	XP_015183663.1	XP_015183663.1 PREDICTED: probable citrate synthase 2, mitochondrial [Polistes dominula]	1	8	8	8	51,836	66668000	121440000	15871000	90552000
XP_015183882.1	XP_015183882.1	XP_015183882.1 PREDICTED: glutathione S-transferase 1-1-like [Polistes dominula]	1	8	8	8	25,034	124830000	622670000	91801000	57415000
XP_015184561.1	XP_015184561.1	XP_015184561.1 PREDICTED: obg-like ATPase 1 [Polistes dominula]	1	8	8	8	44,661	0	67117000	8257100	0
XP_015186089.1;XP_015186079.1	XP_015186089.1;XP_015186079.1	XP_015186089.1 PREDICTED: prosaposin isoform X2 [Polistes dominula];XP_015186079.1 PREDICTED: prosaposin isoform X1 [Polistes dominula]	2	8	8	8	83,057	113850000	0	0	0
XP_015186521.1	XP_015186521.1	XP_015186521.1 PREDICTED: beta-1,3-glucan-binding protein 1-like [Polistes dominula]	1	8	8	8	53,483	0	102270000	23391000	0

XP_015187113.1	XP_015187113.1	XP_015187113.1 PREDICTED: protein yellow-like [Polistes dominula]	1	8	8	8	49,146	102730000	0	0	7357400
XP_015172806.1;XP_015172805.1	XP_015172806.1;XP_015172805.1	XP_015172806.1 PREDICTED: cytochrome c [Polistes dominula];XP_015172805.1 PREDICTED: cytochrome c [Polistes dominula]	2	9	9	9	11,836	60748000	2421300000	931830000	215220000
XP_015174681.1	XP_015174681.1	XP_015174681.1 PREDICTED: cytosol aminopeptidase isoform X2 [Polistes dominula]	1	9	9	9	55,246	31114000	323600000	0	8097100
XP_015174951.1	XP_015174951.1	XP_015174951.1 PREDICTED: triosephosphate isomerase [Polistes dominula]	1	9	9	9	26,906	33044000	77039000	0	279820000
XP_015176841.1	XP_015176841.1	XP_015176841.1 PREDICTED: superoxide dismutase [Mn], mitochondrial [Polistes dominula]	1	9	9	9	24,402	105580000	429930000	34971000	11758000
XP_015177848.1;XP_015182320.1;XP_015182319.1;ACB70872.1;AJG01714.1;AHM24890.1;XP_015172041.1;XP_015172032.1	XP_015177848.1;XP_015182320.1;XP_015182319.1;ACB70872.1;AJG01714.1	XP_015177848.1 PREDICTED: elongation factor 1-alpha [Polistes dominula];XP_015182320.1 PREDICTED: elongation factor 1-alpha-like [Polistes dominula];XP_015182319.1 PREDICTED: elongation factor 1-alpha-like [Polistes dominula];ACB70872.1 elongation factor 1	8	9	9	9	50,304	160930000	83782000	62021000	56640000
XP_015178651.1	XP_015178651.1	XP_015178651.1 PREDICTED: protein disulfide-isomerase [Polistes dominula]	1	9	9	9	56,624	74322000	6698000	0	0
XP_015179473.1;XP_015179472.1;XP_015179471.1;XP_015179470.1	XP_015179473.1;XP_015179472.1;XP_015179471.1;XP_015179470.1	XP_015179473.1 PREDICTED: aldose reductase-like [Polistes dominula];XP_015179472.1 PREDICTED: aldose reductase-like [Polistes dominula];XP_015179471.1 PREDICTED: aldose reductase-like [Polistes dominula];XP_015179470.1 PREDICTED: aldose reductase-like [Pol	4	9	9	9	36,409	11943000	87367000	34347000	16542000
XP_015179484.1	XP_015179484.1	XP_015179484.1 PREDICTED: probable fatty acid-binding protein isoform X2 [Polistes dominula]	1	9	9	2	15,098	0	301910000	124450000	14177000
XP_015180400.1	XP_015180400.1	XP_015180400.1 PREDICTED: translation elongation factor 2 [Polistes dominula]	1	9	9	9	94,719	21210000	13922000	0	8524100
XP_015184783.1	XP_015184783.1	XP_015184783.1 PREDICTED: heat shock protein 83 [Polistes dominula]	1	9	9	5	82,764	101400000	0	0	0
XP_015185192.1;XP_015185191.1;XP_015185190.1	XP_015185192.1;XP_015185191.1;XP_015185190.1	XP_015185192.1 PREDICTED: puromycin-sensitive aminopeptidase isoform X3 [Polistes dominula];XP_015185191.1 PREDICTED: puromycin-sensitive aminopeptidase isoform X2 [Polistes dominula];XP_015185190.1 PREDICTED: puromycin-sensitive aminopeptidase isoform X1	3	9	9	9	104,4	0	157670000	0	2685800
XP_015186243.1	XP_015186243.1	XP_015186243.1 PREDICTED: protein deglycase DJ-1zDJ-1-like [Polistes dominula]	1	9	9	9	23,779	47057000	667040000	82916000	31930000
XP_015189991.1	XP_015189991.1	XP_015189991.1 PREDICTED: protein disulfide-isomerase A3 [Polistes dominula]	1	9	9	9	55,645	0	54288000	11988000	0
XP_015190045.1	XP_015190045.1	XP_015190045.1 PREDICTED: glyoxalase domain-containing protein 4 [Polistes dominula]	1	9	9	9	32,149	127940000	369220000	0	44635000

XP_015191177.1;XP_015191178.1	XP_015191177.1;XP_015191178.1	XP_015191177.1 PREDICTED: dihydropteridine reductase isoform X1 [Polistes dominula];XP_015191178.1 PREDICTED: dihydropteridine reductase isoform X2 [Polistes dominula]	2	9	9	9	25,677	92257000	73082000	41683000	10907000
XP_015171664.1;XP_015171663.1;XP_015171662.1;XP_015171661.1	XP_015171664.1;XP_015171663.1;XP_015171662.1;XP_015171661.1	XP_015171664.1 PREDICTED: dipeptidyl peptidase 3 isoform X3 [Polistes dominula];XP_015171663.1 PREDICTED: dipeptidyl peptidase 3 isoform X3 [Polistes dominula];XP_015171662.1 PREDICTED: dipeptidyl peptidase 3 isoform X2 [Polistes dominula];XP_015171661.1 P	4	10	10	10	83,652	0	129660000	0	0
XP_015171673.1	XP_015171673.1	XP_015171673.1 PREDICTED: isocitrate dehydrogenase [NADP] cytoplasmic [Polistes dominula]	1	10	10	10	53,08	273410000	0	0	28376000
XP_015171840.1;XP_015171838.1	XP_015171840.1;XP_015171838.1	XP_015171840.1 PREDICTED: prolyl endopeptidase isoform X2 [Polistes dominula];XP_015171838.1 PREDICTED: prolyl endopeptidase isoform X1 [Polistes dominula]	2	10	10	10	79,632	2363800	577890000	13019000	0
XP_015176309.1	XP_015176309.1	XP_015176309.1 PREDICTED: vitellogenin-6-like [Polistes dominula]	1	10	10	10	171,21	0	16406000	7826700	24137000
XP_015178346.1;XP_015178343.1;XP_015178341.1;XP_015178339.1;XP_015178335.1;XP_015178347.1;XP_015178344.1;XP_015178340.1;XP_015178336.1;XP_015178338.1	XP_015178346.1;XP_015178343.1;XP_015178341.1;XP_015178339.1;XP_015178335.1;XP_015178347.1;XP_015178344.1	XP_015178346.1 PREDICTED: troponin I isoform X10 [Polistes dominula];XP_015178343.1 PREDICTED: troponin I isoform X8 [Polistes dominula];XP_015178341.1 PREDICTED: troponin I isoform X5 [Polistes dominula];XP_015178339.1 PREDICTED: troponin I isoform X5 [Po	10	10	10	8	23,745	0	14456000	1201400000	0
XP_015178742.1	XP_015178742.1	XP_015178742.1 PREDICTED: uncharacterized protein LOC107067603 [Polistes dominula]	1	10	10	10	225,64	0	201110000	433940000	28595000
XP_015179268.1;XP_015179269.1	XP_015179268.1;XP_015179269.1	XP_015179268.1 PREDICTED: heat shock 70 kDa protein cognate 3 isoform X1 [Polistes dominula];XP_015179269.1 PREDICTED: heat shock 70 kDa protein cognate 3 isoform X2 [Polistes dominula]	2	13	10	10	73,121	100750000	4670500	0	0
XP_015182073.1	XP_015182073.1	XP_015182073.1 PREDICTED: uncharacterized protein LOC107069346 [Polistes dominula]	1	10	10	10	127,62	14907000	851040000	245650000	615190000
XP_015183846.1	XP_015183846.1	XP_015183846.1 PREDICTED: glutathione S-transferase-like [Polistes dominula]	1	10	10	7	23,245	163610000	2662400000	337020000	135570000
XP_015184358.1	XP_015184358.1	XP_015184358.1 PREDICTED: 3-ketoacyl-CoA thiolase, mitochondrial [Polistes dominula]	1	10	10	10	42,023	0	87842000	0	0
XP_015186207.1	XP_015186207.1	XP_015186207.1 PREDICTED: aminoacylase-1-like [Polistes dominula]	1	10	10	10	45,722	0	161680000	47702000	25977000
XP_015187127.1	XP_015187127.1	XP_015187127.1 PREDICTED: uncharacterized protein LOC107072057 [Polistes dominula]	1	10	10	10	21,751	6476900	226280000	15615000	2716500
XP_015173078.1	XP_015173078.1	XP_015173078.1 PREDICTED: protein lethal(2)essential for life-like [Polistes dominula]	1	11	11	11	23,619	283690000	0	7574000	0

XP_015175671.1;XP_015175670.1	XP_015175671.1;XP_015175670.1	XP_015175671.1 PREDICTED: V-type proton ATPase subunit C [Polistes dominula];XP_015175670.1 PREDICTED: V-type proton ATPase subunit C [Polistes dominula]	2	11	11	11	44,406	34948000	79929000	11075000	0
XP_015176906.1;XP_015176908.1;XP_015176907.1	XP_015176906.1;XP_015176908.1;XP_015176907.1	XP_015176906.1 PREDICTED: esterase E4-like [Polistes dominula];XP_015176908.1 PREDICTED: esterase FE4-like isoform X2 [Polistes dominula];XP_015176907.1 PREDICTED: esterase E4-like isoform X1 [Polistes dominula]	3	12	11	11	65,506	0	358680000	0	0
XP_015178252.1	XP_015178252.1	XP_015178252.1 PREDICTED: insulin-like growth factor-binding protein complex acid labile subunit [Polistes dominula]	1	11	11	11	71,434	0	243540000	14333000	0
XP_015178906.1	XP_015178906.1	XP_015178906.1 PREDICTED: delta-1-pyrroline-5-carboxylate dehydrogenase, mitochondrial [Polistes dominula]	1	11	11	11	63,139	121820000	110850000	41089000	0
XP_015185303.1	XP_015185303.1	XP_015185303.1 PREDICTED: vascular endothelial growth factor C [Polistes dominula]	1	11	11	11	36,254	791920000	198370000	41545000	373830000
XP_015186537.1	XP_015186537.1	XP_015186537.1 PREDICTED: nucleoside diphosphate kinase [Polistes dominula]	1	11	11	11	19,679	82621000	3399600000	434920000	147860000
XP_015187088.1;XP_015187087.1	XP_015187088.1;XP_015187087.1	XP_015187088.1 PREDICTED: 3-hydroxyacyl-CoA dehydrogenase type-2-like [Polistes dominula];XP_015187087.1 PREDICTED: 3-hydroxyacyl-CoA dehydrogenase type-2-like [Polistes dominula]	2	11	11	9	27,117	1140800000	244080000	20517000	55781000
XP_015190044.1	XP_015190044.1	XP_015190044.1 PREDICTED: protein lethal(2)essential for life-like [Polistes dominula]	1	11	11	11	27,592	2568900000	229410000	105420000	160290000
XP_015171806.1;XP_015171805.1	XP_015171806.1;XP_015171805.1	XP_015171806.1 PREDICTED: glycerol-3-phosphate dehydrogenase [NAD(+)], cytoplasmic-like isoform X2 [Polistes dominula];XP_015171805.1 PREDICTED: glycerol-3-phosphate dehydrogenase [NAD(+)], cytoplasmic-like isoform X1 [Polistes dominula]	2	12	12	12	38,313	5546100	149810000	6163100	137990000
XP_015172367.1	XP_015172367.1	XP_015172367.1 PREDICTED: malate dehydrogenase, cytoplasmic [Polistes dominula]	1	12	12	12	36,25	90010000	1390000000	353500000	87286000
XP_015173030.1	XP_015173030.1	XP_015173030.1 PREDICTED: acetyl-CoA acetyltransferase, cytosolic [Polistes dominula]	1	12	12	12	41,548	272310000	0	0	0
XP_015176975.1	XP_015176975.1	XP_015176975.1 PREDICTED: ATP synthase subunit beta, mitochondrial [Polistes dominula]	1	12	12	12	55,223	143480000	0	0	0
XP_015177401.1	XP_015177401.1	XP_015177401.1 PREDICTED: chitinase-like protein Idgf4 [Polistes dominula]	1	12	12	12	48,457	5640100	620140000	323360000	12994000
XP_015179142.1;XP_015179141.1;XP_015179139.1	XP_015179142.1;XP_015179141.1;XP_015179139.1	XP_015179142.1 PREDICTED: actin, muscle [Polistes dominula];XP_015179141.1 PREDICTED: actin, clone 205-like [Polistes dominula];XP_015179139.1 PREDICTED: actin, clone 205-like [Polistes dominula]	3	12	12	4	41,757	304930000	344480000	69163000	8611900
XP_015184581.1	XP_015184581.1	XP_015184581.1 PREDICTED: catalase [Polistes dominula]	1	12	12	12	57,659	0	93272000	0	0
XP_015188288.1	XP_015188288.1	XP_015188288.1 PREDICTED: peroxiredoxin 1-like [Polistes dominula]	1	12	12	11	21,569	696240000	142310000	630590000	154700000
XP_015178478.1	XP_015178478.1	XP_015178478.1 PREDICTED: glyceraldehyde-3-phosphate dehydrogenase 2 [Polistes dominula]	1	13	13	13	35,655	99767000	78163000	35786000	246550000

XP_015182129.1;XP_015182128.1;XP_015182127.1;XP_015182126.1;XP_015182125.1;XP_015182124.1;XP_015182123.1	XP_015182129.1;XP_015182128.1;XP_015182127.1;XP_015182126.1;XP_015182125.1;XP_015182124.1;XP_015182123.1	XP_015182129.1 PREDICTED: farnesol dehydrogenase-like isoform X2 [Polistes dominula];XP_015182128.1 PREDICTED: farnesol dehydrogenase-like isoform X2 [Polistes dominula];XP_015182127.1 PREDICTED: farnesol dehydrogenase-like isoform X2 [Polistes dominula];X	7	13	13	13	28,281	3127500000	125860000	212190000	276290000
XP_015184337.1	XP_015184337.1	XP_015184337.1 PREDICTED: cofilin/actin-depolymerizing factor homolog [Polistes dominula]	1	13	13	13	16,832	68794000	3925700000	796260000	228260000
XP_015184835.1	XP_015184835.1	XP_015184835.1 PREDICTED: probable enoyl-CoA hydratase, mitochondrial [Polistes dominula]	1	13	13	13	32,211	2173800000	875590000	60768000	81028000
sp|Q7Z269.1|SP4_POLDO;NP_001310266.1;AAP37412.1	sp|Q7Z269.1|SP4_POLDO;NP_001310266.1;AAP37412.1	sp|Q7Z269.1|SP4_POLDO RecName: Full=Venom serine protease; AltName: Allergen=Pol d 4; Flags: Precursor;NP_001310266.1 venom serine protease precursor [Polistes dominula];AAP37412.1 venom serine protease precursor [Polistes dominula]	3	14	14	14	30,804	26343000000	4189700000	5538900000	9578100000
XP_015172510.1	XP_015172510.1	XP_015172510.1 PREDICTED: probable transaldolase [Polistes dominula]	1	14	14	14	40,533	90646000	1239400000	234610000	32754000
XP_015173711.1;XP_015173710.1;XP_015173709.1;XP_015173706.1;XP_015173708.1	XP_015173711.1;XP_015173710.1;XP_015173709.1;XP_015173706.1;XP_015173708.1	XP_015173711.1 PREDICTED: dihydropyrimidinase isoform X3 [Polistes dominula];XP_015173710.1 PREDICTED: dihydropyrimidinase isoform X3 [Polistes dominula];XP_015173709.1 PREDICTED: dihydropyrimidinase isoform X3 [Polistes dominula];XP_015173706.1 PREDICTED:	5	14	14	14	66,255	0	121010000	41245000	37702000
XP_015174688.1;XP_015174687.1	XP_015174688.1;XP_015174687.1	XP_015174688.1 PREDICTED: heat shock 70 kDa protein cognate 4-like [Polistes dominula];XP_015174687.1 PREDICTED: heat shock 70 kDa protein cognate 4-like [Polistes dominula]	2	25	14	13	70,686	155030000	611450000	123920000	78875000
XP_015174910.1;AEN62318.1	XP_015174910.1;AEN62318.1	XP_015174910.1 PREDICTED: toll-like receptor 8 [Polistes dominula];AEN62318.1 IRP30, partial [Polistes dominula]	2	14	14	14	37,282	1452000000	413800000	109330000	203310000
XP_015174949.1	XP_015174949.1	XP_015174949.1 PREDICTED: 60 kDa heat shock protein, mitochondrial-like [Polistes dominula]	1	14	14	14	60,922	297130000	28943000	0	12420000
XP_015179974.1	XP_015179974.1	XP_015179974.1 PREDICTED: prostaglandin reductase 1-like [Polistes dominula]	1	14	14	14	38,653	1291400000	2654100000	301360000	224240000
XP_015171555.1;XP_015171470.1;XP_015171394.1;XP_015171223.1;XP_015171134.1;XP_015171047.1;XP_015171638.1;XP_015171313.1	XP_015171555.1;XP_015171470.1;XP_015171394.1;XP_015171223.1;XP_015171134.1;XP_015171047.1;XP_015171638.1;XP_015171313.1	XP_015171555.1 PREDICTED: titin isoform X5 [Polistes dominula];XP_015171470.1 PREDICTED: titin isoform X5 [Polistes dominula];XP_015171394.1 PREDICTED: titin isoform X4 [Polistes dominula];XP_015171223.1 PREDICTED: titin isoform X2 [Polistes dominula];XP_0	8	15	15	15	459,79	0	94031000	10414000	26453000

XP_015181184.1	XP_015181184.1	XP_015181184.1 PREDICTED: transcription elongation factor SPT5 [Polistes dominula]	1	15	15	15	219,65	0	395330000	103120000	28090000
XP_015186339.1;XP_015186347.1	XP_015186339.1;XP_015186347.1	XP_015186339.1 PREDICTED: fumarylacetoacetate hydrolase domain-containing protein 2 isoform X1 [Polistes dominula];XP_015186347.1 PREDICTED: fumarylacetoacetate hydrolase domain-containing protein 2A isoform X2 [Polistes dominula]	2	15	15	15	38,607	233320000	734310000	14146000	23991000
XP_015187115.1	XP_015187115.1	XP_015187115.1 PREDICTED: phospholipase A1-like [Polistes dominula]	1	20	15	15	38,417	4942200000	398740000	638950000	433790000
XP_015187970.1;XP_015187969.1	XP_015187970.1;XP_015187969.1	XP_015187970.1 PREDICTED: phosphoglucomutase [Polistes dominula];XP_015187969.1 PREDICTED: phosphoglucomutase [Polistes dominula]	2	15	15	15	61,582	0	1230600000	59463000	104280000
XP_015177608.1;XP_015177607.1;XP_015177606.1	XP_015177608.1;XP_015177607.1;XP_015177606.1	XP_015177608.1 PREDICTED: thioredoxin reductase 1, mitochondrial isoform X3 [Polistes dominula];XP_015177607.1 PREDICTED: thioredoxin reductase 1, mitochondrial isoform X2 [Polistes dominula];XP_015177606.1 PREDICTED: thioredoxin reductase 2, mitochondrial	3	16	16	16	53,688	147900000	538380000	54443000	21668000
XP_015180982.1;XP_015180981.1;XP_015180980.1	XP_015180982.1;XP_015180981.1;XP_015180980.1	XP_015180982.1 PREDICTED: NADP-dependent malic enzyme isoform X3 [Polistes dominula];XP_015180981.1 PREDICTED: NADP-dependent malic enzyme isoform X2 [Polistes dominula];XP_015180980.1 PREDICTED: NADP-dependent malic enzyme isoform X1 [Polistes dominula]	3	16	16	16	63,393	127850000	627440000	94924000	88633000
XP_015184192.1	XP_015184192.1	XP_015184192.1 PREDICTED: dihydrolipoyl dehydrogenase, mitochondrial [Polistes dominula]	1	16	16	16	53,963	76208000	956450000	90920000	129710000
XP_015184667.1	XP_015184667.1	XP_015184667.1 PREDICTED: probable aconitate hydratase, mitochondrial [Polistes dominula]	1	16	16	16	85,762	36729000	10127000	0	120190000
sp|P81656.2|VA5_POLDO;NP_001310265.1;AAT95010.1	sp|P81656.2|VA5_POLDO;NP_001310265.1;AAT95010.1	sp|P81656.2|VA5_POLDO RecName: Full=Venom allergen 5; AltName: Full=Antigen 5; Short=Ag5; AltName: Full=Cysteine-rich venom protein; Short=CRVP; AltName: Allergen=Pol d 5; Flags: Precursor;NP_001310265.1 venom allergen 5 precursor [Polistes dominula];AAT95	3	17	17	17	25,446	35764000000	13009000000	13953000000	17954000000
XP_015171557.1;XP_015171023.1	XP_015171557.1	XP_015171557.1 PREDICTED: glutamyl aminopeptidase-like [Polistes dominula]	2	17	17	17	110,62	0	237630000	34078000	8177200
XP_015175994.1;XP_015176361.1	XP_015175994.1	XP_015175994.1 PREDICTED: aldehyde dehydrogenase X, mitochondrial [Polistes dominula]	2	17	17	17	54,531	0	280830000	35644000	4778600
XP_015176917.1;XP_015176916.1	XP_015176917.1;XP_015176916.1	XP_015176917.1 PREDICTED: heat shock 70 kDa protein cognate 5 [Polistes dominula];XP_015176916.1 PREDICTED: heat shock 70 kDa protein cognate 5 [Polistes dominula]	2	17	17	17	76,051	313350000	194630000	18000000	6859500
XP_015177942.1	XP_015177942.1	XP_015177942.1 PREDICTED: arylphorin subunit beta-like [Polistes dominula]	1	17	17	17	80,459	105510000	8983500000	1640600000	180190000
XP_015181039.1	XP_015181039.1	XP_015181039.1 PREDICTED: stress-induced-phosphoprotein 1 [Polistes dominula]	1	17	17	17	61,013	128090000	25957000	5331100	0

XP_015184999.1;XP_015184998.1;XP_015184997.1;XP_015184995.1;XP_015184994.1;XP_015184992.1;XP_015184996.1	XP_015184999.1;XP_015184998.1;XP_015184997.1;XP_015184995.1;XP_015184994.1;XP_015184992.1;XP_015184996.1	XP_015184999.1 PREDICTED: troponin T, skeletal muscle isoform X3 [Polistes dominula];XP_015184998.1 PREDICTED: troponin T, skeletal muscle isoform X3 [Polistes dominula];XP_015184997.1 PREDICTED: troponin T, skeletal muscle isoform X3 [Polistes dominula];X	7	17	17	17	46,394	0	55325000	1779500000	19455000
XP_015187594.1	XP_015187594.1	XP_015187594.1 PREDICTED: annexin B9-like [Polistes dominula]	1	17	17	17	36,081	18339000	1614900000	55403000	44585000
XP_015187606.1	XP_015187606.1	XP_015187606.1 PREDICTED: uncharacterized protein LOC107072309 [Polistes dominula]	1	17	17	17	101,8	251120000	82336000	0	0
XP_015187119.1	XP_015187119.1	XP_015187119.1 PREDICTED: phospholipase A1-like [Polistes dominula]	1	18	18	18	39,864	27324000000	0	3013100000	52552000
XP_015188027.1	XP_015188027.1	XP_015188027.1 PREDICTED: arginine kinase [Polistes dominula]	1	18	18	18	39,751	256610000	1422300000	473350000	1022600000
XP_015177266.1	XP_015177266.1	XP_015177266.1 PREDICTED: uncharacterized protein LOC107066818 [Polistes dominula]	1	19	19	19	53,574	0	366220000	25599000	4737900
XP_015178295.1	XP_015178295.1	XP_015178295.1 PREDICTED: muscle-specific protein 20 [Polistes dominula]	1	19	19	19	20,58	0	11809000000	5791300000	3102300000
XP_015179722.1	XP_015179722.1	XP_015179722.1 PREDICTED: hyaluronidase [Polistes dominula]	1	19	19	19	42,733	11219000000	7371900000	640100000	3794800000
XP_015187948.1;XP_015187929.1;XP_015187939.1	XP_015187948.1;XP_015187929.1;XP_015187939.1	XP_015187948.1 PREDICTED: uncharacterized abhydrolase domain-containing protein DDB_G0269086-like isoform X3 [Polistes dominula];XP_015187929.1 PREDICTED: uncharacterized abhydrolase domain-containing protein DDB_G0269086-like isoform X1 [Polistes dominula	3	20	20	20	80,894	0	906760000	1167700000	594510000
XP_015172727.1;XP_015172726.1	XP_015172727.1;XP_015172726.1	XP_015172727.1 PREDICTED: malate dehydrogenase, mitochondrial isoform X2 [Polistes dominula];XP_015172726.1 PREDICTED: malate dehydrogenase, mitochondrial isoform X1 [Polistes dominula]	2	21	21	21	32,313	419930000	6440600000	811860000	437520000
XP_015190254.1	XP_015190254.1	XP_015190254.1 PREDICTED: titin-like [Polistes dominula]	1	21	21	21	358,19	0	377930000	65052000	62719000
XP_015184463.1	XP_015184463.1	XP_015184463.1 PREDICTED: fructose-bisphosphate aldolase-like [Polistes dominula]	1	22	22	22	39,587	243280000	1285100000	39969000	1968400000
XP_015176905.1	XP_015176905.1	XP_015176905.1 PREDICTED: esterase E4-like [Polistes dominula]	1	23	23	22	65,577	7552200	3585600000	840770000	78810000
XP_015188978.1;XP_015188979.1	XP_015188978.1;XP_015188979.1	XP_015188978.1 PREDICTED: pyruvate kinase-like isoform X1 [Polistes dominula];XP_015188979.1 PREDICTED: pyruvate kinase-like isoform X2 [Polistes dominula]	2	24	24	24	57,371	54043000	2682000000	565040000	242010000
XP_015177712.1	XP_015177712.1	XP_015177712.1 PREDICTED: heat shock 70 kDa protein cognate 4 [Polistes dominula]	1	28	28	16	71,216	751820000	5677000000	857300000	491210000
XP_015179311.1	XP_015179311.1	XP_015179311.1 PREDICTED: aldose reductase-like [Polistes dominula]	1	29	29	29	69,822	1163700000	1520700000	489390000	321100000
XP_015177945.1	XP_015177945.1	XP_015177945.1 PREDICTED: arylphorin subunit alpha-like [Polistes dominula]	1	30	30	30	81,512	7137500	4385600000	2629400000	119260000

sp|Q6Q250.1|PA13_POLDO;AAS67043.1	sp|Q6Q250.1|PA13_POLDO;AAS67043.1	sp|Q6Q250.1|PA13_POLDO RecName: Full=Phospholipase A1 3; AltName: Allergen=Pol d 1; Flags: Precursor;AAS67043.1 venom phospholipase A1 3 precursor, partial [Polistes dominula]	2	32	32	0	35,019	1,7752E+11	6802200000	21546000000	67721000000
XP_015174448.1;XP_015174447.1;XP_015174446.1;XP_015174445.1	XP_015174448.1;XP_015174447.1;XP_015174446.1;XP_015174445.1	XP_015174448.1 PREDICTED: venom dipeptidyl peptidase 4 [Polistes dominula];XP_015174447.1 PREDICTED: venom dipeptidyl peptidase 4 [Polistes dominula];XP_015174446.1 PREDICTED: venom dipeptidyl peptidase 4 [Polistes dominula];XP_015174445.1 PREDICTED: venom	4	32	32	32	88,858	3762700000	645400000	128320000	41472000
XP_015190339.1	XP_015190339.1	XP_015190339.1 PREDICTED: vitellogenin-like [Polistes dominula]	1	33	33	33	201,78	108420000	227340000	161260000	46806000
XP_015175448.1;XP_015175447.1;XP_015175446.1;XP_015175445.1;XP_015175444.1;XP_015175443.1;XP_015175450.1	XP_015175448.1;XP_015175447.1;XP_015175446.1;XP_015175445.1;XP_015175444.1;XP_015175443.1;XP_015175450.1	XP_015175448.1 PREDICTED: filamin-A isoform X4 [Polistes dominula];XP_015175447.1 PREDICTED: filamin-A isoform X3 [Polistes dominula];XP_015175446.1 PREDICTED: filamin-A isoform X2 [Polistes dominula];XP_015175445.1 PREDICTED: filamin-A isoform X1 [Poliste	7	37	37	5	244,4	81708000	1163900000	459950000	312630000
XP_015171927.1	XP_015171927.1	XP_015171927.1 PREDICTED: transferrin [Polistes dominula]	1	42	42	42	79,341	31985000	13059000000	2107400000	124540000
XP_015183297.1	XP_015183297.1	XP_015183297.1 PREDICTED: twitchin isoform X8 [Polistes dominula]	1	43	43	23	937,43	0	582950000	463310000	462960000
XP_015191559.1	XP_015191559.1	XP_015191559.1 PREDICTED: nuclear anchorage protein 1-like [Polistes dominula]	1	52	52	52	694,62	13260000	956000000	174350000	138400000
XP_015184655.1	XP_015184655.1	XP_015184655.1 PREDICTED: apolipophorins [Polistes dominula]	1	111	111	111	377,7	200680000	2985600000	10125000	51857000
XP_015191788.1	XP_015191788.1	XP_015191788.1 PREDICTED: titin [Polistes dominula]	1	129	129	129	1594,7	0	1102200000	1876900000	1598000000
